# Cultural attraction in pottery practice: Group-specific shape transformations by potters from three communities

**DOI:** 10.1093/pnasnexus/pgae055

**Published:** 2024-02-27

**Authors:** Tetsushi Nonaka, Enora Gandon, John A Endler, Thelma Coyle, Reinoud J Bootsma

**Affiliations:** Graduate School of Human Development and Environment, Kobe University, Kobe 657-8501, Japan; Institute of Archaeology, University College London, London WC1H 0PY, UK; Centre for Integrative Ecology, School of Life and Environmental Sciences, Deakin University, Waurn Ponds, VIC 3216, Australia; College of Science & Engineering, James Cook University, Cairns, QLD 4878, Australia; Institute of Movement Sciences, Aix Marseille University, CNRS, F-13288 Marseille cedex 09, France; Institute of Movement Sciences, Aix Marseille University, CNRS, F-13288 Marseille cedex 09, France

**Keywords:** cultural evolution, pottery, cultural attraction, biased transformation, skill

## Abstract

Pottery is a quintessential indicator of human cultural dynamics. Cultural alignment of behavioral repertoires and artifacts has been considered to rest upon two distinct dynamics: selective transmission of information and culture-specific biased transformation. In a cross-cultural field experiment, we tested whether community-specific morphological features of ceramic vessels would arise when the same unfamiliar shapes were reproduced by professional potters from three different communities who threw vessels using wheels. We analyzed the details of the underlying morphogenesis development of vessels in wheel throwing. When expert potters from three different communities of practice were instructed to faithfully reproduce common unfamiliar model shapes that were not parts of the daily repertoires, the morphometric variation in the final shape was not random; rather, different potters produced vessels with more morphometric variation among than within communities, indicating the presence of community-specific deviations of morphological features of vessels. Furthermore, this was found both in the final shape and in the underlying process of morphogenesis; there was more variation in the morphogenetic path among than within communities. These results suggest that the morphological features of ceramic vessels produced by potters reliably and nonrandomly diverge among different communities. The present study provides empirical evidence that collective alignment of morphological features of ceramic vessels can arise from the community-specific habits of fashioning clay.

**Significance Statement**

In archaeology, ceramic analysis has been concerned with categorizing types according to vessel shape to describe cultural dynamics at specific periods. Nevertheless, using morphological features of artifacts to reveal cultural dynamics is not trivial, because what gives rise to social patterning of morphological features of ceramic artifacts is not well understood. In a cross-cultural experiment, we show that when potters from three cultural communities reproduced the same model shapes that were not part of their daily repertoires, morphological features of ceramic vessels reliably and nonrandomly diverged among different communities through the dynamic process of fashioning clay. This suggests the collective alignment of morphological features of ceramic vessels can arise from the community-specific habits of pottery practice.

## Introduction

Different human groups exhibit specific variations in their artifacts and techniques, including tools (1), materials (2), methods of manufacture (3), and decoration styles (4). These aspects have changed over time at various scales. However, there is a limited understanding of how the collective alignment of artifacts and techniques develops and stabilizes across generations. A quintessential case is the emergence and maintenance of pottery tradition—the craft of making container objects out of clay. Pottery requires a complex sequence of tasks, including the selection of raw materials, preparation of clay paste, fashioning, and firing, which brings into question how pottery is made and diverges among populations in societies past and present (5). A recent study, for example, has shown that in Europe around the sixth millennium BC, the resemblance among sites in pottery morphology, decoration, and technique remained notable even at distances of up to 500 km, indicating knowledge of pottery production was somehow shared by groups located geographically far apart (6). Another interesting case is the later development of the wheel-throwing technique in pottery that exploits the fast-rotating wheel, whose introduction dates back to around the second millennium BC (7). The wheel-throwing technique allows the production of even and symmetrical ceramic vessels in a matter of minutes, speeding up production times, yet it simultaneously increases the difficulty and lengthens the learning process for the skills involved in pottery production (5). In wheel-throwing, the plastic deformation of clay emerges through a dynamic interplay of physical forces (8–11). Presumably owing to a dramatic shift in skills and habits, very diverse assemblages of pottery across sites in the eastern Mediterranean are observed during the Bronze Age when the wheel technique appeared, indicating an uneven picture of adoption and resistance (12, 13).

In cultural evolution research, there has been a theoretical divide between approaches that highlight the selective transmission of information (14–16) and those that emphasize shared cultural attractors (or biased transformation) shaping the persistent, nonrandom variation in artifacts and individual behavior (17–19). Imitation has been proposed as a particularly important process among those involved in the transmission of information (20, 21). According to this view, stability at the macro level of the culture is considered to rest upon the fidelity at the micro level of interindividual copying of information. Once the required condition of high-fidelity copying is met, the information transmission approach allows quantitative modeling that predicts the distribution of frequencies of traits in different choice scenarios (e.g. prestige-biased transmission, conformist-biased transmission) (22–24). Cultural attraction, in contrast, involves a process where variations that occur in their recurrence tend to exhibit certain directional transformations specific to the communities of practice. Within this view, there are biasing factors present in a populated environment which affect the direction toward which behavior of members of the community unfold over time. When such biasing factors are shared in a population, community-specific variation of artifacts can persist robustly even without micro-level fidelity copying of a model product. It is important to note, however, that these two distinct dynamics are not mutually exclusive (25). It is possible that community-specific biased attention inherent in pottery techniques provides the source of variation that is selectively copied by subsequent generations.

In archaeology, ceramic analysis has been concerned with the issue of what gives rise to the social patterning of the individual practice of pottery production (26). The two aforementioned approaches—information transmission and cultural attraction—provide different scenarios for the source of community-specific variation in pottery tradition. The first approach models innovations (or mutations) such as changes in the shape of vessels as small, undirected copying errors. In this approach, the source of community-specific variation is the selective accumulation of nondirected variations. The second approach, in contrast, focuses on the biases present in the behavior of potters. In the second approach, the central question becomes whether community-specific morphological variation of vessels reliably arises from the systematic deviation of products at each recurrence. In principle, such community-specific patterns of transformation can arise not only from cognitive biases but also from the individual artisans’ whole modus vivendi, including their unique way of assessing the situation, structure of the environment that emphasizes specific affordances, regularities of behavior of other cooperating individuals, and so on (27).

In this study, to uncover the detailed dynamics underlying the community-specific variation of ceramic artifacts, we report a cross-cultural experiment with professional potters from three different cultural backgrounds (French and two Indian communities—Hindu and Muslim). We examined the morphological changes in the wheel-thrown clay body, starting from its initial preformed stage after centering and opening operations, and continuing up to the point of reaching the final form. The experiment was designed to test directly whether pottery-making behavior would reliably result in nonrandom divergence of morphogenetic paths resulting in community-specific morphological features of the vessels. In the present experiment, 21 professional potters were instructed to reproduce four unfamiliar model shapes, which were not part of the daily repertoire in their communities of practice. The same models were given to the three groups of potters. Within-individual and within-community random variation of morphology arising from the reproduction of the model was used as a null hypothesis to test against. This allowed us to obtain independent evidence for the following hypotheses: (i) if community-specific morphological variation of vessels reliably arises from the systematic deviation in the process of shaping, then there should be nonrandom, culturally specific deviation in morphogenetic path as well as in the final shape when potters reproduce value-neutral shapes, where the morphological path and final shape would be more similar to the ones exhibited by potters from one's cultural group than a random path and shape; and (ii) if the source of cultural bias is idiosyncratic practice of potters, then throughout the forming process, the degree of among-potter variation should be greater than within-potter variation within each community, and this pattern should be stronger during the behavioral process of morphogenesis compared with that in the final shape. If the above two hypotheses turn out to be true, then it follows that a nonrandom source of morphological variation resides in the behavior of potters, which can give rise to a community-specific deviation of morphological traits.

The field experiment addressing these issues took place in three distinct pottery workshops—one in France (Bourgogne) and the other two in India (Bulandshahar district, Uttar Pradesh). In India, one workshop belonged to the Hindu community (group Prajapati) and the other to the Muslim community (group Multani Kumhar) which both live and work in the same village. In the workshops where potters regularly work, video-based data acquisition was conducted. Working in their usual conditions, potters were asked to reproduce the four shapes (referred to as cylinder, bowl, sphere, and vase) presented in pictures using two different quantities of clay (0.75 and 2.25 kg) without any indication of the required dimension (Table 1). The potters were simply instructed to faithfully replicate the model shape and throw vessels with the thinnest walls possible. Nine French potters, six Prajapati potters, and six Multani Kumhar potters participated in the experiment. All 21 participating potters were confirmed experts, each having >10 years of experience in wheel-throwing. Among the four model shapes, vase is not typical of classic ceramic forms and is a very unfamiliar shape for all three cultural groups. In addition, the vase presents the highest mechanical stress and, therefore, the highest throwing difficulty, which increases with the amount of clay used (9). Therefore, in the following analyses, we concentrated most of our attention on the vase thrown with 2.25 kg of clay by all participating potters.

**Table 1.** The four nontraditional forms of vessels (cylinder, bowl, sphere, and vase) which potters reproduced with two different quantities of clay (0.75 and 2.25 kg) and means and SDs of height (*H*) and maximal diameter (MD) of final vessel forms and throwing duration across trials for the eight vessel types thrown by the potters in the three communities of practice.

Type	Mass of clay (kg)	Prajapati			Multani Kumhar			French		
		*H* (cm)	MD (cm)	Duration (s)	*H* (cm)	MD (cm)	Duration (s)	*H* (cm)	MD (cm)	Duration (s)
Cylinder 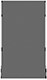	0.75	17.27 (0.81)	8.86 (0.73)	110.17 (28.72)	15.64 (1.69)	10.39 (0.45)	81.99 (22.09)	17.91 (1.60)	11.19 (0.76)	76.45 (14.57)
	2.25	26.36 (2.00)	11.16 (0.94)	166.42 (31.69)	25.47 (1.73)	14.26 (0.79)	114.91 (36.23)	28.94 (3.03)	15.57 (1.00)	131.99 (23.86)
Bowl 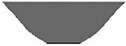	0.75	7.29 (0.76)	20.63 (0.69)	71.23 (17.24)	6.36 (0.62)	21.82 (1.41)	32.69 (10.66)	9.35 (1.06)	22.28 (1.84)	74.19 (26.79)
	2.25	10.49 (1.09)	29.59 (1.08)	146.8 (53.77)	10.11 (0.83)	31.87 (1.83)	62.24 (21.26)	13.84 (1.62)	34.20 (2.47)	135.58 (38.17)
Sphere 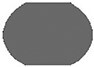	0.75	12.01 (0.98)	14.70 (0.63)	119.61 (50.75)	11.08 (1.21)	14.88 (0.86)	75.78 (24.45)	12.28 (1.30)	16.69 (0.82)	105.60 (32.75)
	2.25	18.13 (1.41)	20.74 (0.86)	169.90 (59.72)	17.02 (1.63)	21.97 (0.82)	100.03 (29.92)	19.04 (2.26)	24.62 (1.41)	173.10 (53.77)
Vase 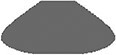	0.75	10.54 (1.25)	15.60 (0.97)	127.45 (47.47)	9.60 (0.74)	15.62 (0.86)	78.50 (22.20)	10.43 (1.69)	17.25 (0.88)	103.62 (38.49)
	2.25	15.71 (2.00)	22.30 (0.85)	206.43 (78.31)	14.53 (0.94)	23.34 (0.94)	97.34 (29.16)	15.61 (2.44)	25.33 (1.09)	155.81 (53.99)

## Results

### Comparison of morphogenesis across communities and individuals

**Fig. 1.**
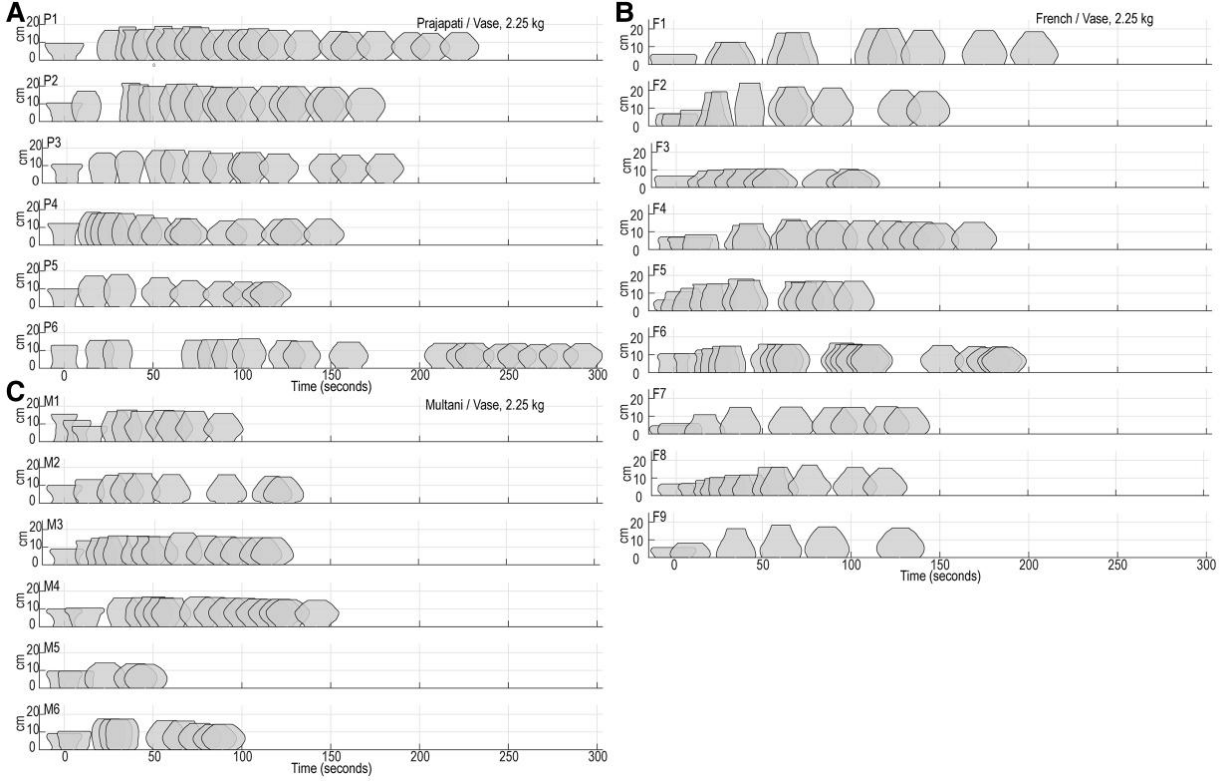
Morphological development as a function of time for the last of the five trials of producing vase using 2.25 kg of clay thrown by each of the 21 potters. A) Prajapati potters, B) French potters, and C) Multani Kumhar potters. Successive outlines on the timelines represent the vessel form after each fashioning gesture of the potter, from the initial preformed shape (*t* = 0) up to the final vessel shape. The size scale (height) is indicated on the *y*-axis.

To capture the morphological development leading to the final form, we digitized the outline of each thrown vessel from video frames after every clay-deforming manual fashioning gesture. Figure 1 shows clay form as a function of time for the last of the five trials of vase (2.25 kg) produced by each of the 21 potters (see Figs. S1–S7 for the development of other form types). As can be seen from Fig. 1, there were idiosyncratic differences among potters and communities of practice in morphological development toward each final vessel form. For example, at the onset of the forming phase (*t*0 in Fig. 1), the preforms produced by the initial gestures of Prajapati potters (except P6) and Multani Kumhar potters tended to exhibit a wide-rimmed aperture. Subsequently, the morphogenetic paths taken by the potters diverged at the second forming gesture: In Prajapati potters, a lip around the aperture was lost, and the shapes of the vessels invariably reached elongated barrel-like shapes taller than the final products after the second forming gesture, prior to reaching the shorter and wider final shape that had a sharp curve at the height where the diameter is maximal (Fig. 1A). In Multani Kumhar potters, the elongation of the shape was more gradual and less pronounced where the intermediate shapes were not much taller than the final products, and the paths to the final shape were quite distinct among the potters (Fig. 1C). French potters initially prepared disk-like, flat preforms (except F6), and proceeded to the shapes that had the widest part at the very bottom (Fig. 1B). Reflecting such a morphogenetic path, the vessels produced by French potters tended to have wider bottoms compared with those produced by Prajapati and Multani Kumhar potters.

On average, the time it took for the Multani Kumhar potters to reach the final form was shorter compared with Prajapati and French potters (Table 1). Final vessels produced by Multani Kumhar potters tended to have smaller absolute dimensions (i.e. thicker walls) compared with other groups. Mixed-effects model ANOVA found that throwing duration was community specific for all vessel types (*F* > 3.82, *P* < 0.05) except sphere (0.75 kg; *F* = 3.54, *P* = 0.05), where Multani Kumhar consistently spent the shortest time to fashion vessels except cylinder (0.75 kg; Table 1). Despite the same quantity of clay being used to produce unfamiliar shapes, the final products of morphogenetic processes varied in dimension across potters in different communities. Mixed-effects model ANOVA found a significant effect of community on absolute maximal width for all form types (*F* > 11.43, *P* < 0.001) except Bowl (0.75 kg; *F* = 2.51, *P* = 0.11), where French potters consistently produced vessels with the greater absolute maximal diameter (and thinner walls) compared with Prajapati and Multani Kumhar potters. Mixed-effects model ANOVA also found a significant effect of community on height for cylinder and bowl (*F* > 5.25, *P* < 0.05), where Multani Kumhar potters consistently produced vessels with lesser height compared with the other two groups (Table 1). Within individual potters of the three communities, the morphogenetic processes (i.e. the morphological development), as well as final products, were fairly consistent across the five trials (see supplementary materials for the other trials).

**Fig. 2.**
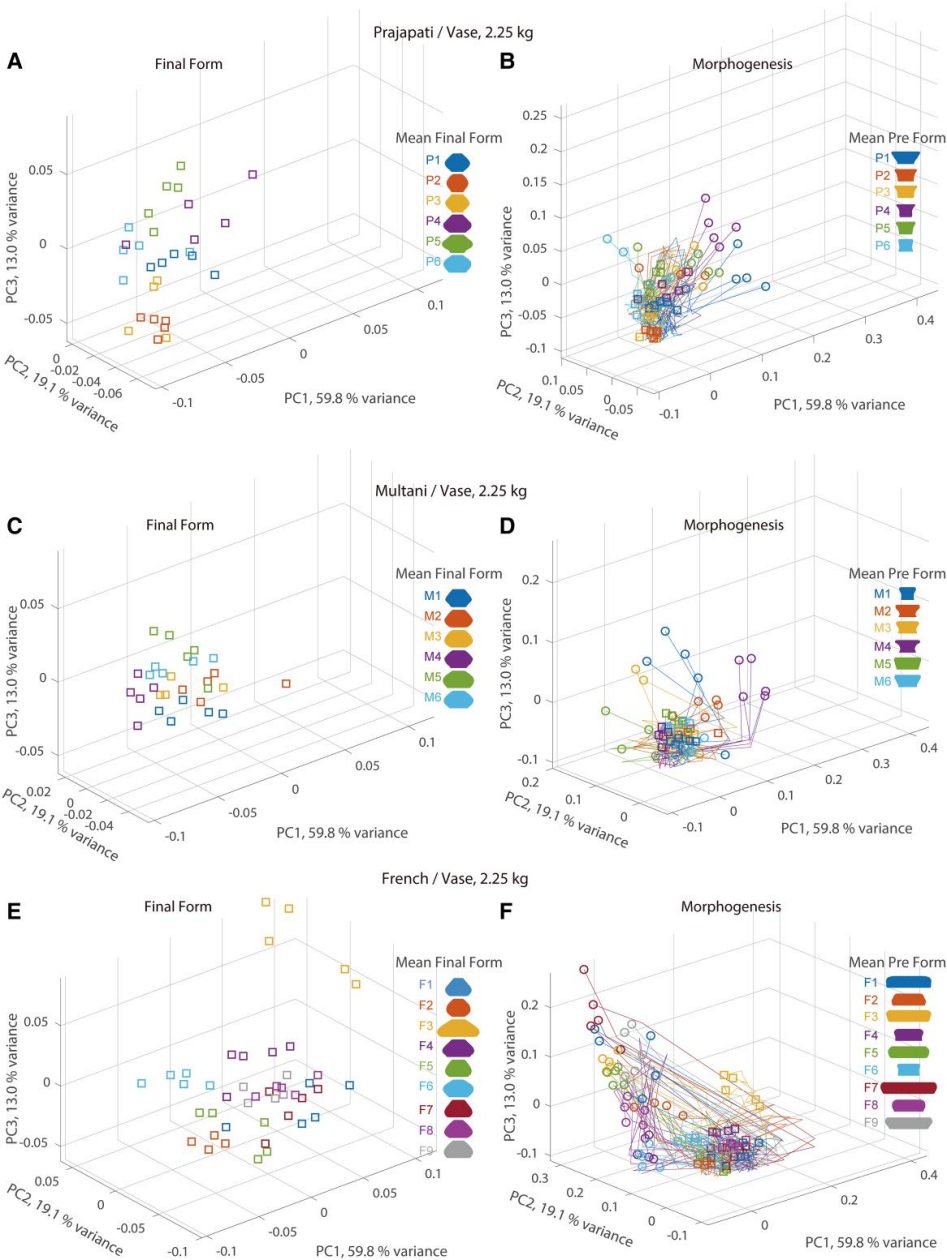
Development of the morphology of vase (2.25 kg) in shape space. Right panels: Development of vessel morphology is represented as trajectories through 3D shape space, from the initial preformed shape (open circles) to the final shape (open squares), for vessels thrown by B) six Prajapati potters, D) six Multani Kumhar potters, and F) nine French potters. Individual potters are color-coded. For each potter, mean initial shape (preform) is depicted on the right side. Left panels: Zoom on final vessel shapes (open squares) thrown by A) Prajapati potters, C) Multani Kumhar potters, and E) French potters. For each potter, the mean final form is depicted on the right side.

To quantify the morphological variation of vessels independent of their size, we conducted elliptical Fourier analysis on all digitized clay outlines. The resulting series of 30 pairs of Fourier coefficients was normalized with respect to the first pair of coefficients to correct for size differences so pure shapes could be analyzed (28). The full set of size-corrected Fourier coefficients was then subjected to a principal component (PC) analysis. For all evolving vessels, over 90% of the total shape variance was captured by the first three PCs, based on which a 3D “shape space” was constructed (Fig. 2). In this shared 3D shape space, we traced the evolution of shape, facilitating both qualitative (visual inspection) and quantitative (numerical) comparisons to assess similarities and differences in shape.

The five final shapes of vase (2.25 kg) thrown by each of the 21 potters are presented in the left panels of Fig. 2 (see Figs. S8–S14 for the other vessel types). For the final shapes produced by 21 potters, permutation tests (29) revealed statistically significant heterogeneity among individuals as well as among communities for all the vessel types (Table 2, “Final shape” column). These results demonstrate that different potters produced vessels with more morphometric variation among than within potters, and that potters from different communities produced vessels with more morphometric variation among than within communities. Thus, expert potters imprinted subtle but identifiable individual signatures as well as community signatures even on the nontraditional, unfamiliar types of vessels which the potters had not been habitually producing (see (10, 30–33) for the analysis of traditional vessels).

**Table 2.** Results of the permutation tests performed on the size-corrected coefficients, resulting from elliptical Fourier analyses of the final clay shapes and the morphogenetic spaces (that reflect the morphogenetic paths taken by the potters) for each vessel type thrown by the French (nine potters), Prajapati (six potters), and Multani Kumhar potters (six potters).

			Final shape		Morphogenetic space	
Type	Component	*df*	*F*	*P*	*F*	*P*
Cylinder, 0.75 kg	Community	2	6.95	<0.0001	9.43	<0.0001
	Individual	18	4.83	<0.0001	12.06	<0.0001
Cylinder, 2.25 kg	Culture	2	6.80	<0.0001	6.17	<0.0001
	Individual	18	5.34	<0.0001	20.93	<0.0001
Bowl, 0.75 kg	Community	2	12.42	<0.0001	14.98	<0.0001
	Individual	18	7.87	<0.0001	9.58	<0.0001
Bowl, 2.25 kg	Community	2	5.25	<0.001	9.43	<0.001
	Individual	18	10.49	<0.0001	17.32	<0.0001
Sphere, 0.75 kg	Community	2	5.12	<0.001	9.32	<0.0001
	Individual	18	5.57	<0.0001	10.42	<0.0001
Sphere, 2.25 kg	Community	2	4.79	<0.001	7.65	<0.0001
	Individual	18	5.70	<0.001	18.12	<0.0001
Vase, 0.75 kg	Community	2	4.63	<0.001	5.05	<0.001
	Individual	18	7.48	<0.0001	12.66	<0.0001
Vase, 2.25 kg	Community	2	5.69	<0.001	12.99	<0.0001
	Individual	18	8.79	<0.0001	14.80	<0.0001

For each vessel type, within-potter effects are based on five trials.

Individual- and community-specific shape differences appeared not only in the final products but were also present in morphological paths (toward the final shape) during the shaping phase. As can be seen from the right panels in Fig. 2, the trajectories in the 3D shape space (corresponding to the shape development during the forming phase) varied markedly over potters as well as over communities when shaping the vase (2.25 kg); the same phenomenon was observed for the other nontraditional vessel types (Figs. S8–S14). To estimate the intergroup and interindividual variation in morphogenesis, we conducted the following two analyses. First, we modeled the morphogenetic path in the common 3D shape space (i.e. temporal variation of PC1, PC2, and PC3) using generalized additive modeling (GAM) (34). Here, we compared the model with a single global smoother for all observations (model G), the model with a community-specific smoothers with a shared penalty (model S), and the model having a community-specific smoothers plus random effects for individual-level intercepts and temporal variations (model SI). For all vessel types, Akaike's information criterion (AIC) values indicate that model S provided a better fit than model G, and model SI provided a better fit than model S (Table S1). When we trained the model with data from odd-number trials (i.e. trials 1, 3, and 5) to predict data from even-number trials (i.e. trials 2 and 4), model S better predicted out-of-sample data than model G, and model SI predicted out-of-sample data better than model S for all vessel types (Table S1 and Figs. S15–S22). Taken together, these results provide strong evidence that there are intercommunity as well as interpotter variations in morphogenetic path in the 3D shape space.

**Fig. 3.**
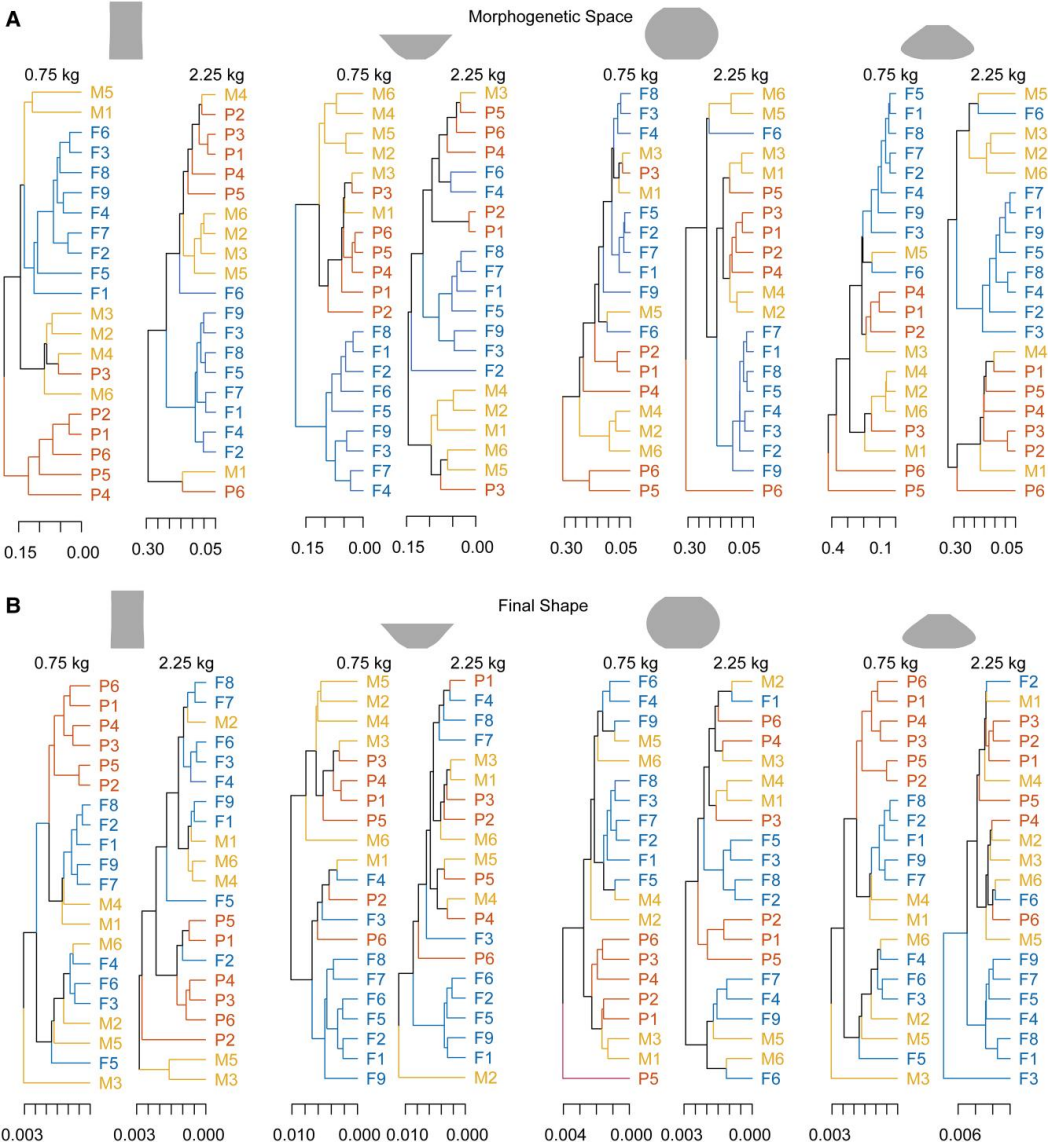
Dendrograms based on the between-potter similarity of the final shape (A) and of the morphogenetic subspace (shape space) built from trials from each potter producing each vessel type (B). The distance of the divergence between the branches is indicated in the horizontal axis. The further right the diverging point is, the more similar the vessel shapes and morphogenetic subspace in the corresponding branches. The letter before the number indicates the potter's community (P: Prajapati potters; M: Multani Kumhar potters; F: French potters).

In the second analysis, we performed PCA on the 30 pairs of (i.e. 60D) Fourier coefficient dataset from each trial, and then computed cross-projection similarity between pairs of 3D shape subspaces of different trials (35, 36). Permutation tests based on the dissimilarity matrix (i.e. distance matrix) obtained from the cross-projection similarity (see Methods for details) confirmed that, for each of the nontraditional types thrown, among-potter variation in morphogenetic space was significantly larger than within-potter variation, and that among-community variation in morphogenetic path was significantly larger than within-community variation (see Table 2, “Morphogenetic space” column). This result confirmed that the potters exhibited individual- and community-specific ways of fashioning the vessels, even when they were shaping nontraditional vessels that were unfamiliar to them. To further visualize the effect of community on the reproduction of unfamiliar vessel types, we constructed dendrograms based on the shape dissimilarity matrix (Fig. 3). The dendrograms indicate that the potters have community-specific ways of shaping the vessels, which leaves distinguishable traits in the shapes of the final products.

### Comparison of the range of morphological variation at different phases of the throwing process

**Fig. 4.**
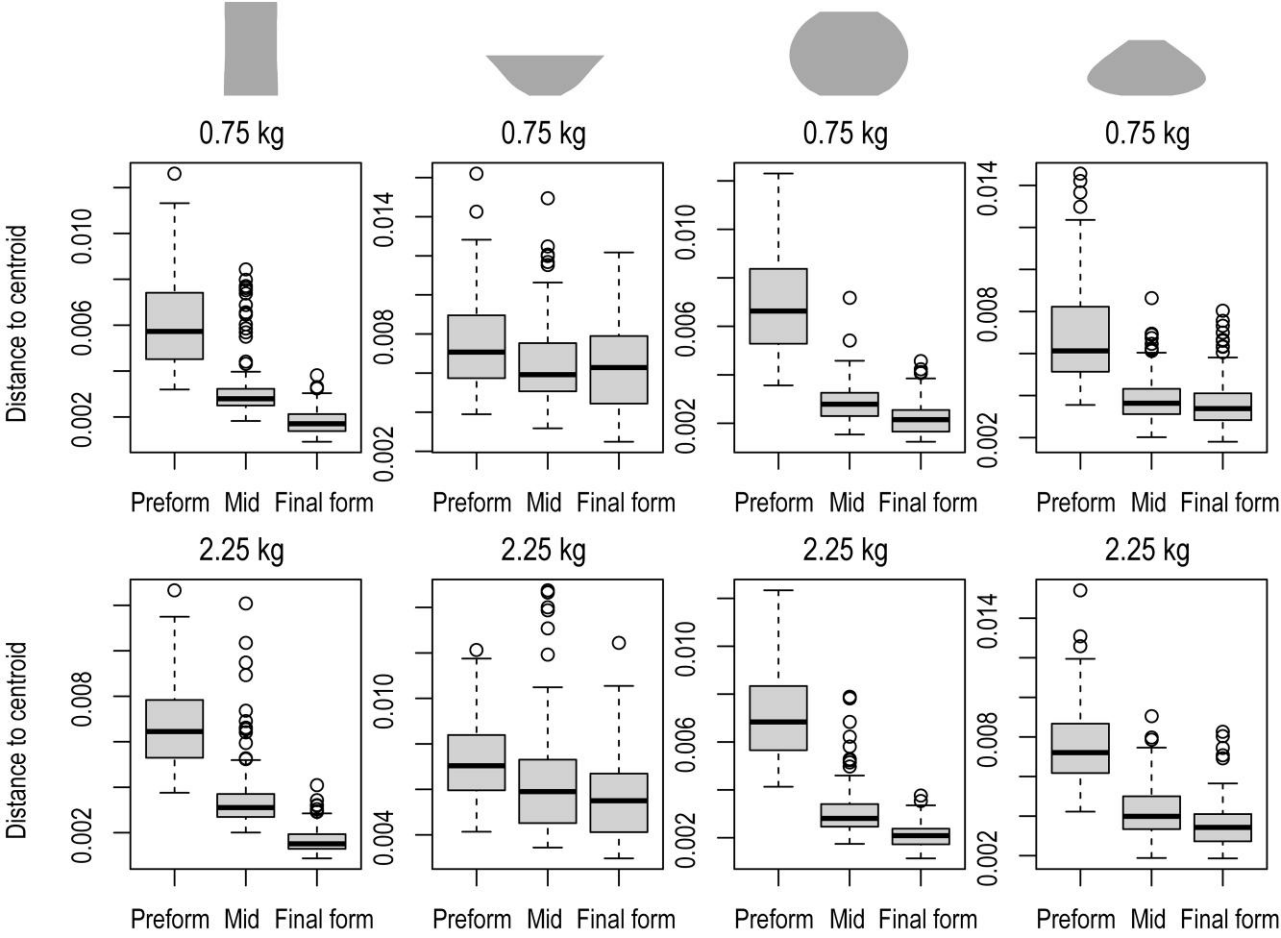
Multivariate heterogeneity of morphometric characteristics at the initial preformed stage, the middle stage, and the final stage for eight model types. Box plots represent Euclidean distances from individual points in the full dimensional space of size-corrected Fourier coefficients to their group centroid at the three stages. Data from all 21 potters are pooled. The solid lines in the box indicate the medians of the data.

Visual inspection of the right panels in Fig. 2 for the vase (2.25 kg) indicates that the starting positions of the trajectories (corresponding to the shape products of the preforming phase) varied markedly over potters. Yet, although potters started from considerably different locations in the shape space, they generally converged toward closely neighboring shape-space locations at the final stage (left panels of Fig. 2). To further uncover the multivariate heterogeneity of the morphometric characteristics of the shapes at different stages of the shaping process, we conducted the *betadisper* multivariate dispersion analysis (37) on the set of Fourier coefficients at three stages (preform, middle point of the morphogenetic trajectory, and final product) across all trials. This analysis confirmed that the degree of heterogeneity of the morphometric characteristics of the clay significantly differed across the three stages (Table S2), and that the range of variation in the morphological characteristics generally narrowed successively toward the final shape (Fig. 4), with the exception of the bowl (0.75 kg) which had a similar range of variation between middle and final stages.

**Fig. 5.**
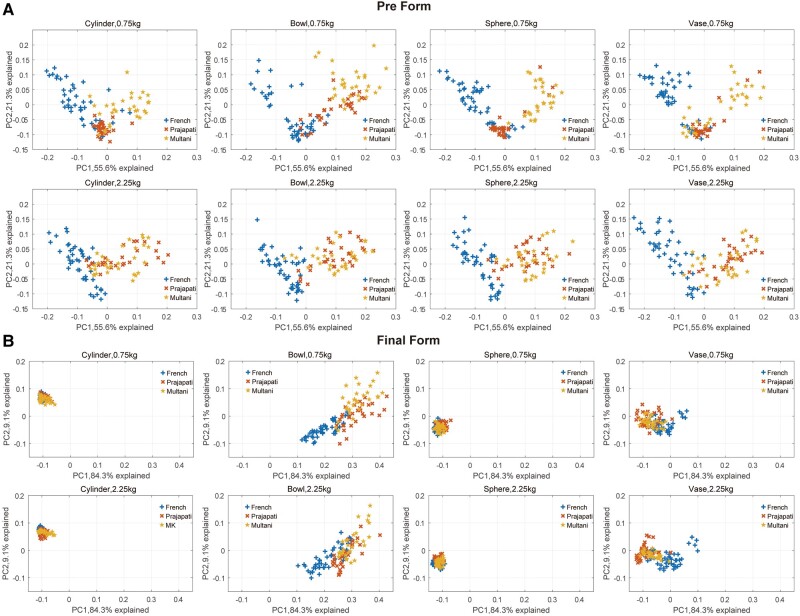
Morphological distribution of A) preform and B) final form of the eight vessel types thrown by French (blue), Prajapati (red), and Multani Kumhar (yellow) potters. Shape space was constructed based on the two PCs of the PC analysis conducted separately for the preform and the final form (i.e. shape space is not common between the preform and final form). The points in the shape space are based upon elliptical Fourier coefficients derived for every pot shape which show the variations among three communities of practice.

Visual inspection of Fig. 1 suggests that the manner of preforming the clay prior to shaping (*t*0 in Fig. 1) was different across potters and across communities of practice. The above results confirmed that idiosyncrasies in morphological routes (toward the final shape) appeared not only at intermediary stages but may have already been present before the onset of the forming phase. To visualize the effect of community on the starting positions of the morphological trajectories (corresponding to the profile immediately following the preforming phase of centering and opening), we subjected the size-corrected Fourier coefficients from the initial and final stages separately to the PC analysis and constructed the separate shape spaces that correspond to initial and final stages using the first two PCs (Fig. 5). The comparison between the distribution of the shape products of the preforming phase (top panels of Fig. 5) and that of the final vessel shape (bottom panels of Fig. 5) revealed that there were community-specific distribution patterns in the shape products of the preforming phase that were almost identical across various vessel types whose final shapes were entirely different. Permutation tests on the set of Fourier coefficients of the preforms (i.e. the products of the preforming phase prior to the shaping phase) from all vessel types confirmed that across community variation in preformed clay shape (all vessel types pooled) was significantly larger than within community variation (*df* = 2, *F* = 16.08, *P* < 0.0001), and that among potter variation in preformed clay shape from all vessel types was significantly larger than within potter variation (*df* = 2, *F* = 18.59, *P* < 0.0001). This result suggests that the expert potters have common individual- and community-specific starting positions for morphological trajectories even when they intend to shape very different, unfamiliar vessel types.

## Discussion

In the present study, we asked whether community-specific morphological variation of vessels would reliably arise from the systematic deviation in the process of pottery making, which could potentially lead to group-level stability of morphological features. We conducted cross-cultural field experiments that specifically tested whether there would be nonrandom, community-specific deviation in the morphogenetic path as well as in the final shape when potters reproduce value-neutral shapes. When expert potters from three different communities were instructed to faithfully reproduce unfamiliar model shapes that are not parts of the daily repertoires, it turned out that morphometric variation in the final shape was not random; rather, different potters produced vessels with more morphometric variation among than within communities, indicating the presence of community-specific deviations of morphological features of vessels. Furthermore, this was found both in the final shape and during the underlying process of shaping (morphogenesis); there was more variation in the morphogenetic path among than within communities. These results demonstrate that the morphological features of vessels produced by potters reliably and nonrandomly diverge among different communities of practice, owing to the cultural biases present in the dynamic process of fashioning clay.

We further tested whether the source of such community bias lies in the idiosyncratic habits of practice of potters within each community. Our analysis of the heterogeneity of morphogenesis among individuals found that throughout the forming process, the degree of among-potter variation was greater than within-potter variation, and this pattern was stronger in the process of transformation compared with that in the final shape, with the degree of heterogeneity being greatest at the initial stage of the shaping phase. Even though there was a common transformational invariant (i.e. a style of change) underlying the morphogenetic paths taken by the members of the same community of practice, significant degrees of individual difference were present within each community, where individual potters followed clearly distinctive, idiosyncratic routes through morphological space toward the less variable final shape. Evolution is dependent upon variation regardless of its source. We have presented direct evidence for variation among individual potters and communities which easily allows evolutionary divergence in pottery shape at various levels and degrees.

In our experiment, the 21 potters demonstrated idiosyncratic fashioning styles to flexibly cope with the task of reproducing unfamiliar shapes, which nevertheless did not compromise the socially patterned nature of the practice of pottery making. Such co-existence of the idiosyncrasy of fashioning styles of individual potters and community-specific habits of practice seems to call for renewed consideration of the relation between individual development of skill and attention shared within the community in the process of making. Expertise in complex skills of handicrafts such as pottery wheel-throwing involves not merely the ability to exhibit stereotypical behavior, but also the ability to tailor one's behavior in such a way to resonate with the task-relevant aspects of the environment (38, 39). In such skills, learning is not entirely reducible to the imitation of someone else's behavioral solution, because imitated behavior is indifferent to the changing econiche in which artisans are embedded. To achieve a wide range of purposes in a dynamic context requires learning how to actively explore the constraints and opportunities offered by the environment that change continuously, without which stable intergenerational recurrence of community-specific handicrafts would not have been realized.

As some authors rightly pointed out (19, 40), the description of humans in cultural evolutionary theories has often been very “thin”—they imitate the behavior of others, which could be biased in a few simple ways, plus random mutations. But in reality, a mental library of behavioral solutions acquired by faithful imitation of others’ behavior may not be viable, simply because each one of us lives with a unique body-in-an-environment. By highlighting potter idiosyncrasies in vessel morphogenesis which are socially patterned, our study adds to the growing realization that we should explore theories that assign greater complexity to individual humans, as this is necessary for providing thorough explanations of skill learning and craftsmanship (41–47). We further stress that the factors that affect group-specific habits of practice need not be confined within human brains, but they can be distributed throughout the whole system of relations constituted by the presence of a person with others in her/his ordinary environment, which relate indirectly to the reliable canalization of the development and behavior of individuals under normal circumstances (40). In this view, seemingly contradictory results of the co-existence of idiosyncratic fashion styles by individual potters and community-specific deviations of morphological features of vessels may not be contradictory after all, but rather an inevitable outcome arising from the underlying mechanism of sharing of embodied skills in an eventful environment.

Regarding the flexibility to tailor one's behavior in such a way to resonate with the task-relevant aspects of the immediate environment, we found that each potter had his own preferred preformed shape (i.e. the products of the preforming phase) which subsequently diverges and evolves flexibly into different final shapes. We also found that the range of individual variation in the morphology of clay is the largest at this initial stage. This result further implies that the interindividual morphological differences in the finished artifacts do not linearly translate to the interindividual differences in the processes involved in producing the same artifacts: While the finished artifacts may be selected toward a specific form (related to a community standard, market value, etc.), the process of making might be selected favoring flexibility which retains the pluripotency of clay to evolve into a wide range of forms. On their way to the goal, artisans meet various combinations of social, ecological, and functional demands that are specific to different phases of fashioning, and as the artisans make their way through, these demands, in turn, are likely to manifest themselves as different ranges of variation at different stages of morphogenesis, which may have implications for understanding the variation of morphological features observed in ceramic assemblages.

We demonstrated that community divergence of morphological features of ceramic vessels can arise from the process of fashioning clay in which shapes emerge over time, whose invariant patterns exhibit subtle directionality specific to communities of practice. Even when unfamiliar shapes without any explicit payoffs are being reproduced, expert potters reliably exhibit their behavioral repertoires that bear individual and community signatures while flexibly coping with novel goals. Such individual- and community-specific patterns underlying the transformative process of clay shape are likely to bias the range of morphological variation of the vessels to provide a directional effect, which would result in nonrandom variation of morphological features shared within the community of practice.

## Materials and methods

### Experimental design

We conducted a series of field experiments in order to evaluate between-culture as well as between-potter variation during morphogenesis when professional traditional potters threw unfamiliar vessel types. The study involved the participation of 21 professional potters representing three distinct cultural groups: 9 were French potters (group FR, 7 males and 2 females, mean age 51.1 ± 7.1 years old), while the remaining 12 consisted of 6 Indian male potters from the Hindu community (group Prajapati, abbreviated PR, 6 males, mean age 41.3 ± 14.9 years old) and 6 from the Muslim community (group Multani Kumhar, abbreviated MK, 6 males, mean age 33.7 ± 4.5 years old). All the potters in the study were right-handed. French (FR) potters utilized electrical wheels activated by a pedal, while Indian potters from the Hindu community (PR) employed high-inertia flywheels launched with a wooden stick. Potters from the Muslim community in India (MK) utilized foot-operated, low-inertia kick-wheels. Upon receiving adequate information, individual potters made voluntary decisions to participate in the study. They were financially compensated for their participation. All potters provided written, informed consent before taking part in the study. The study was carried out in accordance with the ethical standards of the Declaration of Helsinki and was approved by the University of Aix-Marseille (EG's affiliation at the time).

The field experiments occurred under their typical conditions of practice. Potters from each workshop were instructed to faithfully replicate four distinct model shapes with the thinnest walls possible using two different quantities of clay (0.75 or 2.25 kg), leading to a total of eight experimental conditions (Table 1). Pictures of the four shapes (referred to as cylinder, bowl, sphere, and vase, respectively) were presented without indicating the specific dimensions to be replicated. The four shapes were not included in the daily repertoire of any of the potters. Each potter crafted 5 specimens for each of the 8 vessel types, resulting in a total production of 40 vessels per potter. Throughout the experiment, the order of the various conditions was randomized within each block of eight trials to prevent any systematic learning effects. The day before the experiment, potters practiced the task by crafting at least one vessel under each of the eight experimental conditions. Under standardized conditions, the experimental sessions were captured on video using a Panasonic NV-GS320 camcorder. The camera was secured on a tripod with lens orientation centered on the vertical rotation axis of the wheel. The camera was set at a height of 30 cm above the wheel's level, positioned at a horizontal distance of 4–6 m. The center of the wheel was aligned with the lower edge of the video scene. At the beginning of each recording, the zoom was adjusted to completely cover a calibration object (inverted T-shape) measuring 36 cm in height and 42 cm in width, positioned on the wheel.

For each trial, images of the clay body profile following each fashioning gesture were extracted from the video frames (image resolution: 720 × 576 pixels; video sampling frequency: 25 fps). The first image captured the profile right after the (centering and opening) preforming phase, while the last image captured the final profile. The intervening images documented the intermediate profiles during the development of the form. These sequential profiles documented the morphogenesis of the vessel. The total duration of the forming process was also examined. From the images, we extracted the 2D coordinates of the right half of the cross-sectional profiles by tracing them on a Cintiq 21UX Wacom (Kazo, Japan) tablet with an integrated screen. We conducted all subsequent analyses using MATLAB (MathWorks, Natick MA, USA). Using a calibration factor obtained from the digitized dimensions of the calibration object, the profile coordinates were transformed from pixels to centimeters. We re-sampled the profiles to generate an equal number of points (256) at regular intervals along the vertical (*y*) axis. The resulting coordinates were then smoothed using a low-pass filter. As wheel-thrown vessels are typically axisymmetric, we converted the profiles to complete pot outlines. This was achieved by multiplying the horizontal (*x*) coordinates by −1 to create the corresponding left edge. We measured the dimensions of the vessel in terms of height and maximum diameter. Of the total 840 vessels thrown (21 potters, each throwing 5 specimens of 8 different vessel types), 12 vessels could not be analyzed due to problems with the video-recording. To quantify the shape of each vessel, we applied an elliptical Fourier transformation to its outline (28). To account for size differences, we normalized the resulting series of pairs of coefficients with respect to the first coefficients (28). A PC analysis was applied to the resulting set of 30 size-corrected Fourier coefficient pairs. When all trials were pooled, over 90% of the full-dataset (8,168 outlines) total variance was captured by the first three components (59.8, 19.1, and 13.0%, respectively), in which each particular shape could thus be represented as a point in a 3D PC space, allowing comparisons of shape similarities and differences. To further quantify the similarity in the morphogenesis, the 30 pairs of (i.e. 60D) size-corrected Fourier coefficients for each trial were individually analyzed using PC analysis to calculate cross-projection similarity (35, 36). For this, we first calculated the total variance accounted for by the first three PCs of the morphogenetic path of one trial (V1). Then, we projected the morphogenetic path from the trial onto the first three PCs of another trial (V2) and calculated the total variance explained. Finally, we computed the ratio V2/V1, which approaches 1 to the extent that the second subspace resembles the first. As this measure is not necessarily symmetric, we computed the ratio V2/V1 in both directions and reported the average ratio as an index of subspace similarity, which reflects the similarity of the morphogenetic paths between trials. The index of subspace similarity (S) can be converted to the index of subspace dissimilarity (D) by computing 1-S, based on which the dissimilarity matrix of all the trials was obtained.

### Statistical analysis

For all statistical tests, the (two-sided) alpha level used was 0.05. To examine statistical differences among shapes, nested permutation tests (30) were conducted on the 30 pairs of size-corrected Fourier coefficients using the *nested.npmanova* function in the *R* package *BiodiversityR* (48). This analysis employed a two-level hierarchical model, where individual potters were nested within cultural groups. This analysis, conducted during the final stages of shape development, examined the heterogeneity of shapes among the potters and cultural groups within shape types. If the test yielded significant results, it would indicate the presence of individual or cultural influences on shape. In addition, using the dissimilarity matrix obtained through cross-projection similarity analysis, we performed the separate nested permutation tests that tested for heterogeneity of morphogenetic paths among the potters and the cultural groups within shape types, whose statistical significance indicates the presence of individual or cultural influences on the processes of morphogenesis. Based on the dissimilarity matrix across potters for each of the eight shape classes, we constructed dendrograms presenting the similarities among the morphogenetic paths taken by the potters as well as those among the final shapes (Fig. 3). If cultural influences were at play, we would observe a distinct pattern in the dendrograms, highlighting differences among the branches representing various cultural groups. Using the *R* function *hclust*, dendrograms were generated based on the unweighted pair-group mean arithmetic method.

Morphogenetic paths in the common 3D shape space were modeled using GAM with the R package *mgcv* (49). We modeled PC1, PC2, and PC3 separately with a single global smoother for all observations as a function of time (model G), with a community-specific smoothers as a function of time with a shared penalty (model S), and the model having a community-specific smoothers as a function of time with a shared penalty plus random effect smoothers for both individual potter and time to account for individual-level temporal variation (model SI). Time has been transformed into percent time, where the start of the shaping phase (i.e. the end of the preforming phase) is 0 and the end of the shaping phase is 100. AIC scores were used to compare the model fit. AIC is an estimate of relative expected of Kullback-Leibler (K-L) distance from each model *g_i_* to unknown process *f* that generated the observed data (50). To further enable evaluation of out-of-sample performance, we split the data into testing and training sets. As there are multiple trials of data, we used data from the odd trials (i.e. trials 1, 3, and 5) to fit (train) models, and the even trials (i.e. trials 2 and 4) to test the fit. We calculated the total deviance of the out-of-sample data that we had previously held out. The deviance is equal to two times the sum of the difference between the log-likelihood of the out-of-sample data as predicted by each model.

The heterogeneity of the morphometric characteristics of the vessels at different stages of the fashioning process was evaluated by a multivariate dispersion analysis on the set of Fourier coefficients at three stages (preform, middle point of the morphogenetic trajectory, and final product) across all trials, using the *betadisper* function in the R package *vegan* (51). To test the presence of individual- and community-specific preferred starting positions of morphological routes, a separate permutation test was conducted on the size-corrected Fourier coefficient scores of preformed clay shapes where data from all vessel types were pooled. For permutation tests, dendrograms, and dispersion tests, the alternative-Gower distance was used as a dissimilarity measure (37), and 10,000 randomizations were used to obtain *P*-values.

## Supplementary Material

pgae055_Supplementary_Data

## Data Availability

The data that support the findings of this study are openly available in Dryad at https://doi.org/10.5061/dryad.9ghx3ffpv.
