# Supplementary material for: Cultural attraction in pottery practice: Group-specific shape transformations by potters from three communities
Source: PNAS Nexus. 2024 Feb 27;3(2):pgae055. doi: 10.1093/pnasnexus/pgae055 (PMC10898857; doi:10.1093/pnasnexus/pgae055)
Supplement: pgae055_Supplementary_Data [file pgae055_supplementary_data.pdf]

PNAS nexus

# Supplementary Information for

## Cultural attraction in pottery practice: Group-specific shape transformations by potters from three communities

Tetsushi Nonaka[1*], Enora Gandon[2], John A. Endler[3,4], Thelma Coyle[5], Reinoud J. Bootsma[5]

[1] Graduate School of Human Development and Environment, Kobe University, Kobe, 657-8501, Japan

[2] Institute of Archaeology, University College London, London, WC1H 0PY, United Kingdom

[3] Centre for Integrative Ecology, School of Life & Environmental Sciences, Deakin University, Waurn Ponds, VIC 3216, Australia

[4] College of Science & Engineering, James Cook University, Cairns, QLD 4878, Australia

[5] Institute of Movement Sciences, Aix Marseille University, CNRS, F-13288 Marseille cedex 09, France

* Corresponding author: Tetsushi Nonaka

**Email:** tetsushi@people.kobe-u.ac.jp

**Author Contributions:** E.G., R.J.B., and T.N. designed research; E.G. conducted field experiments; E.G. and T.C. processed the data; T.N. and J.A.E. and analyzed the data; T.N. wrote the manuscript. E.G., R.J.B., T.C., and T.N. reviewed and edited the manuscript. T.N. and E.G. contributed equally to this work.

**Competing Interest Statement:** Authors declare that they have no competing interests.

**Classification:** Social and Political Sciences, Anthropology

**Keywords:** cultural evolution, pottery, cultural attraction, biased transformation, skill

**This PDF file includes:**

Figures S1 to S22
Tables S1 to S2

## Figures and Tables

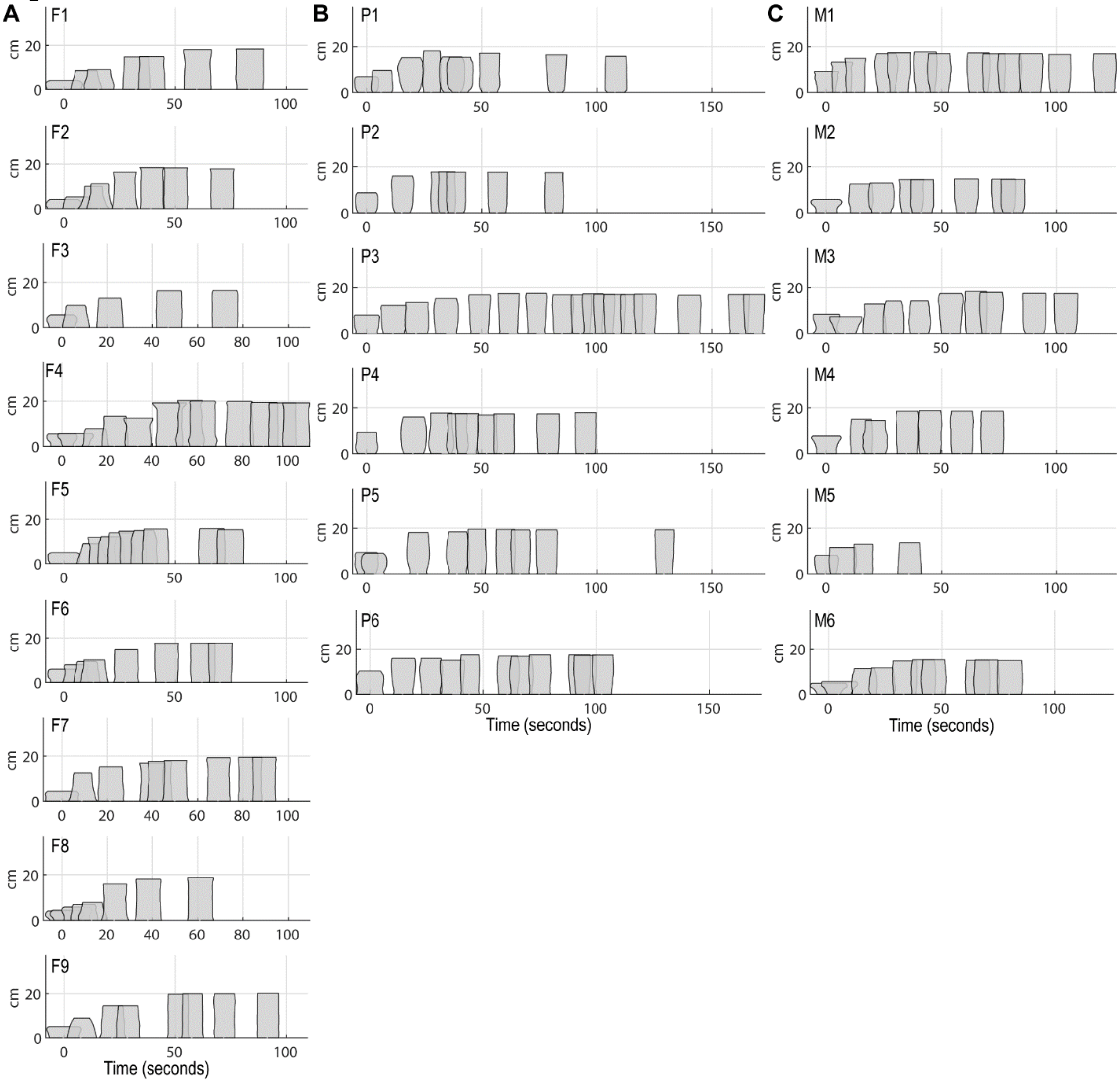


**Figure S1.** Morphological development as a function of time for the last of the five trials of producing Cylinder using 0.75 kg of clay thrown by each of the 21 potters. (A) Prajapati potters, (B) French potters, and (C) Multani Kumhar potters. Successive outlines on the timelines represent the vessel form after each fashioning gesture of the potter, from the initial pre-formed shape ($t$ = 0) up to the final vessel shape. The size scale (height) is indicated on the y-axis.

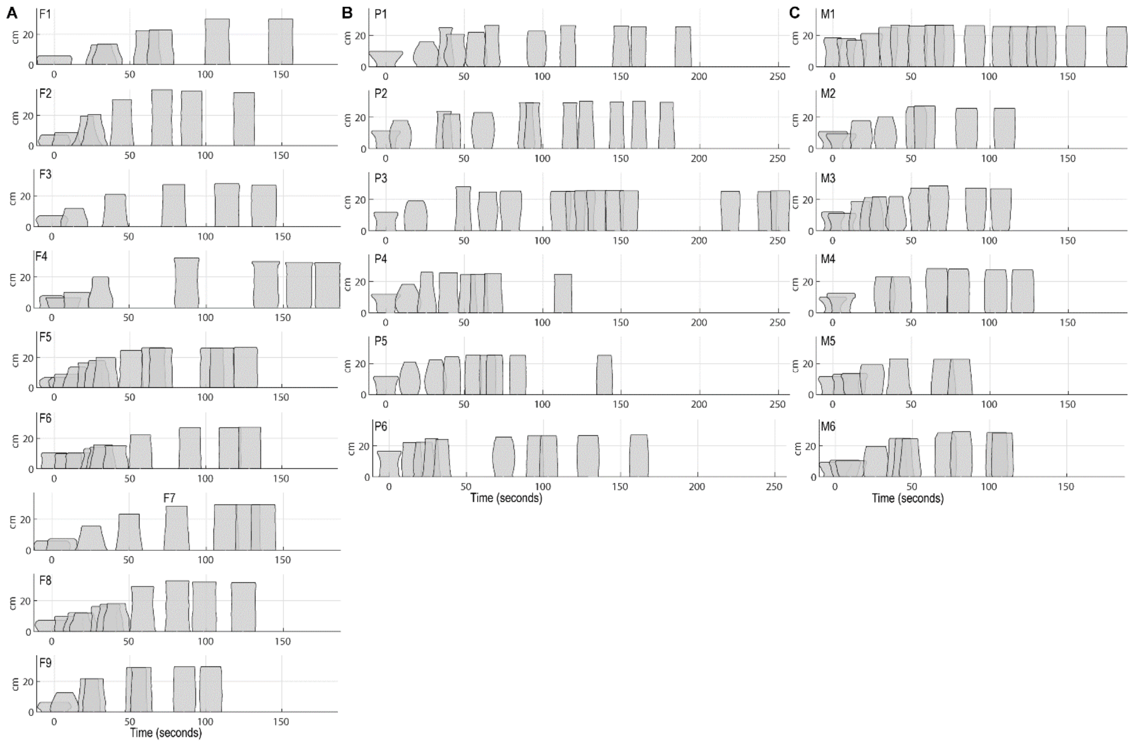


**Figure S2.** Morphological development as a function of time for the last of the five trials of producing Cylinder using 2.25 kg of clay thrown by each of the 21 potters. (A) Prajapati potters, (B) French potters, and (C) Multani Kumhar potters. Successive outlines on the timelines represent the vessel form after each fashioning gesture of the potter, from the initial pre-formed shape (*t* = 0) up to the final vessel shape. The size scale (height) is indicated on the y-axis.

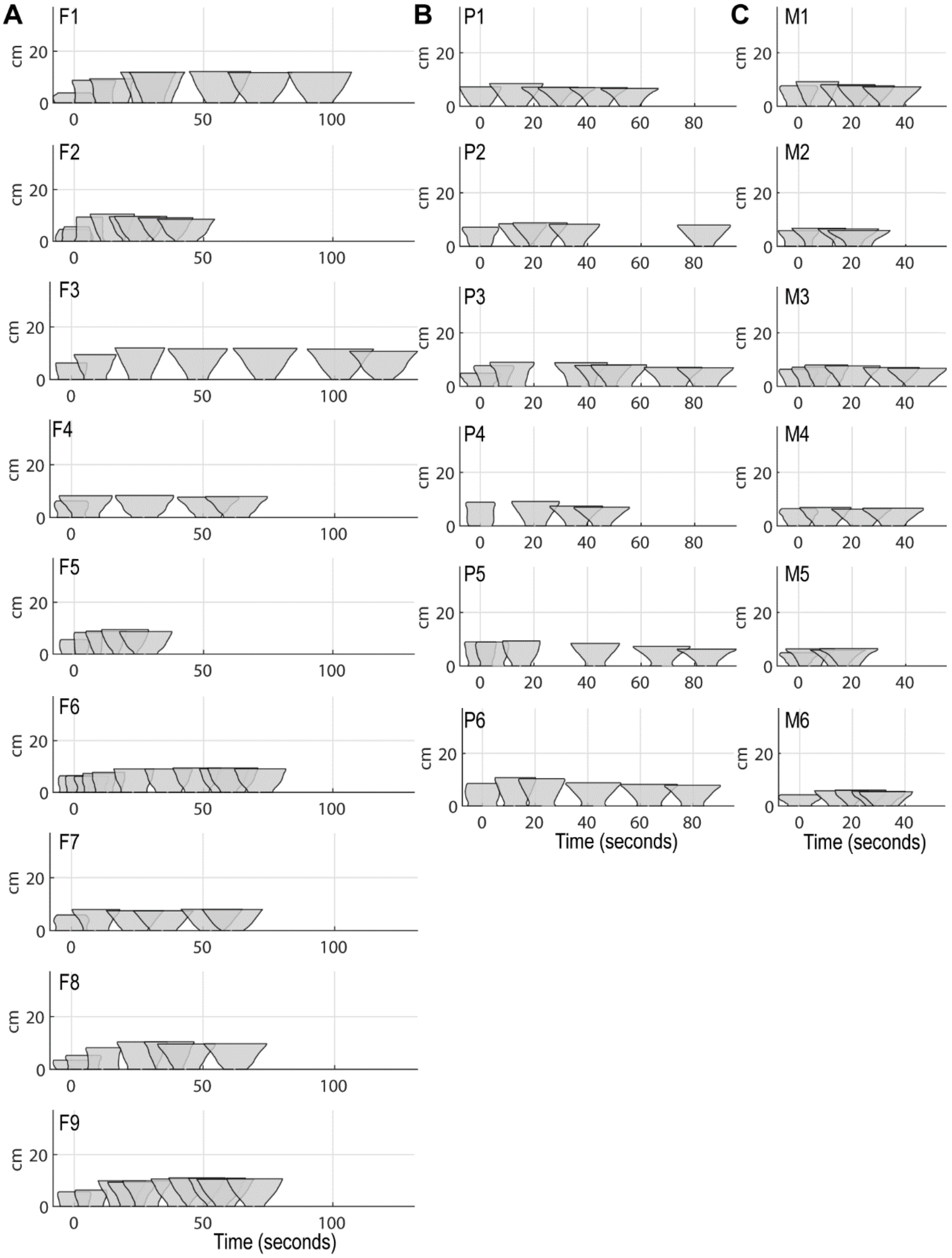


**Figure S3.** Morphological development as a function of time for the last of the five trials of producing Bowl using 0.75 kg of clay thrown by each of the 21 potters. (A) Prajapati potters, (B) French potters, and (C) Multani Kumhar potters. Successive outlines on the timelines represent the vessel form after each fashioning gesture of the potter, from the initial pre-formed shape ($t$ = 0) up to the final vessel shape. The size scale (height) is indicated on the y-axis.

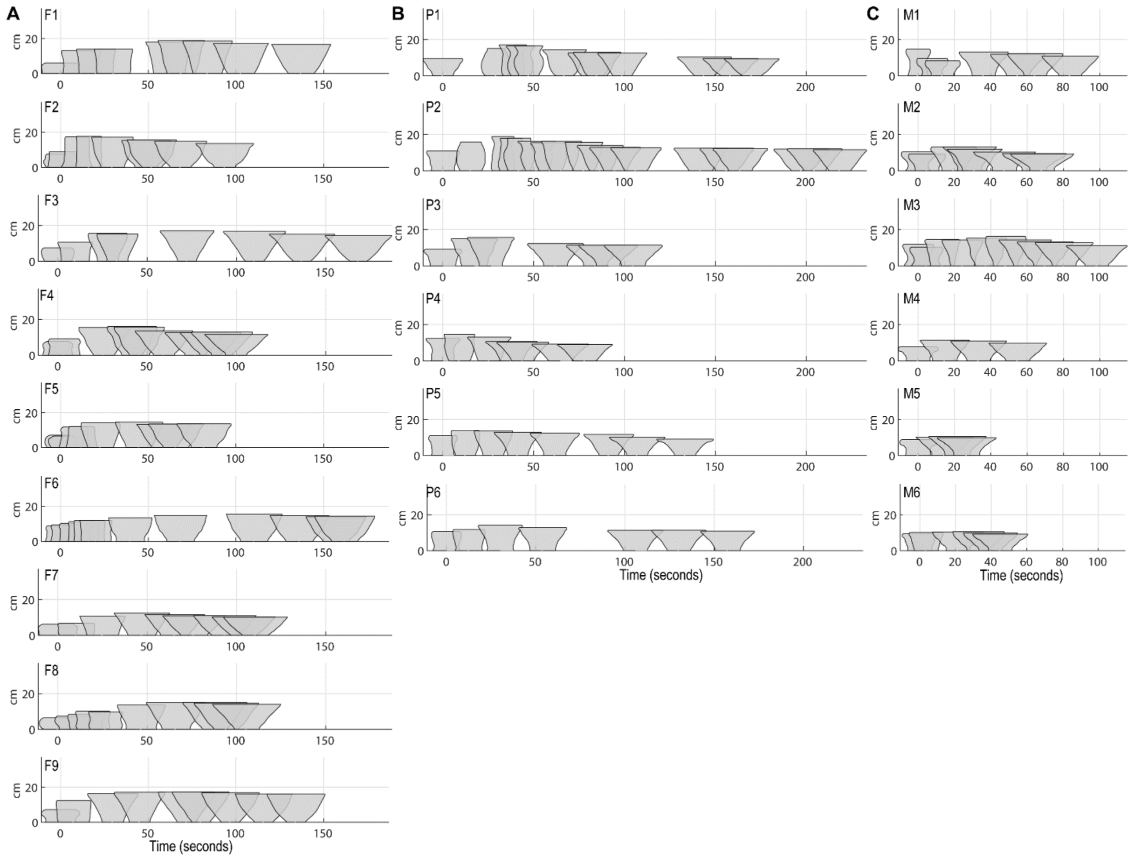


**Figure S4.** Morphological development as a function of time for the last of the five trials of producing Bowl using 2.25 kg of clay thrown by each of the 21 potters. (A) Prajapati potters, (B) French potters, and (C) Multani Kumhar potters. Successive outlines on the timelines represent the vessel form after each fashioning gesture of the potter, from the initial pre-formed shape (*t* = 0) up to the final vessel shape. The size scale (height) is indicated on the y-axis.

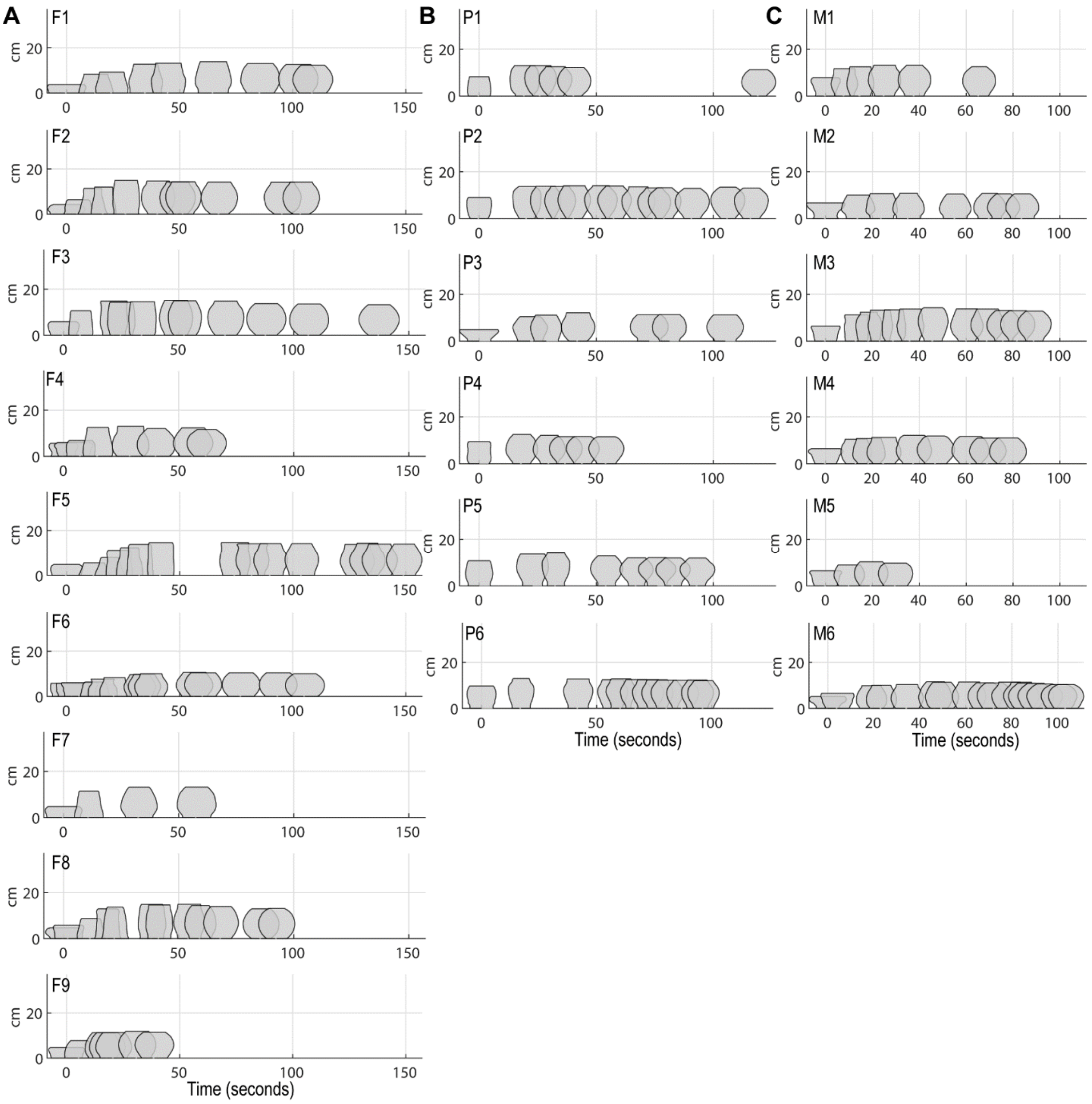


**Figure S5.** Morphological development as a function of time for the last of the five trials of producing Sphere using 0.75 kg of clay thrown by each of the 21 potters. (A) Prajapati potters, (B) French potters, and (C) Multani Kumhar potters. Successive outlines on the timelines represent the vessel form after each fashioning gesture of the potter, from the initial pre-formed shape (*t* = 0) up to the final vessel shape. The size scale (height) is indicated on the y-axis.

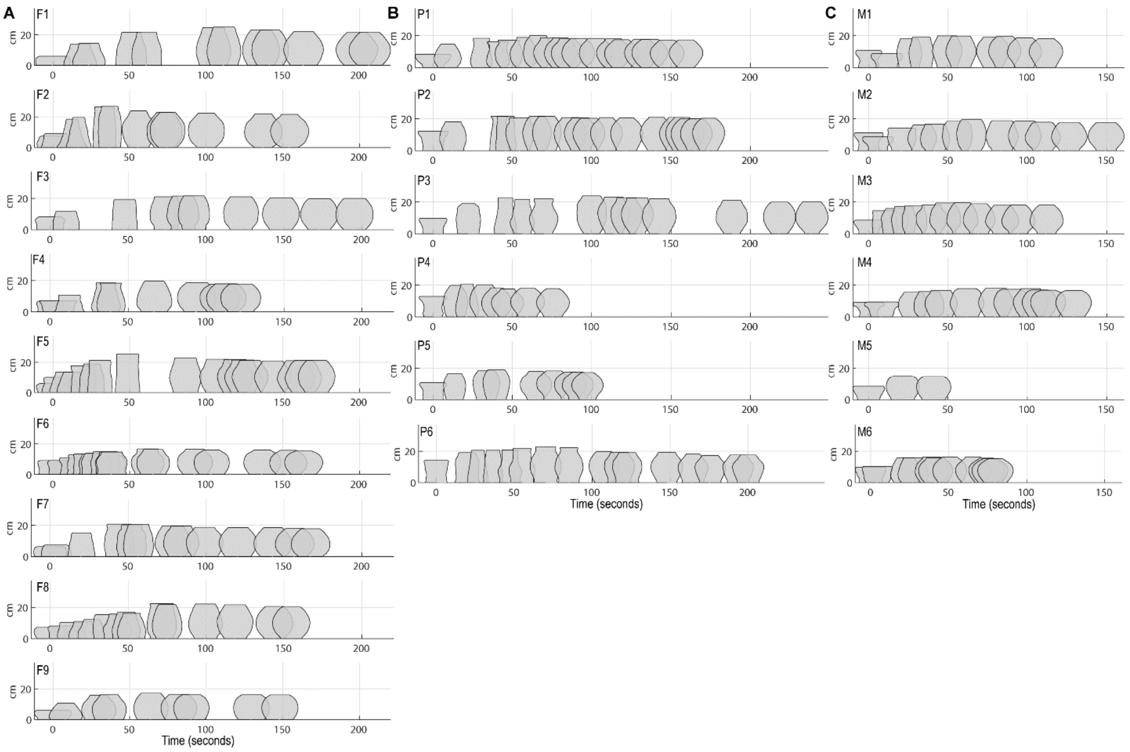


**Figure S6.** Morphological development as a function of time for the last of the five trials of producing Sphere using 2.25 kg of clay thrown by each of the 21 potters. (A) Prajapati potters, (B) French potters, and (C) Multani Kumhar potters. Successive outlines on the timelines represent the vessel form after each fashioning gesture of the potter, from the initial pre-formed shape ($t$ = 0) up to the final vessel shape. The size scale (height) is indicated on the y-axis.

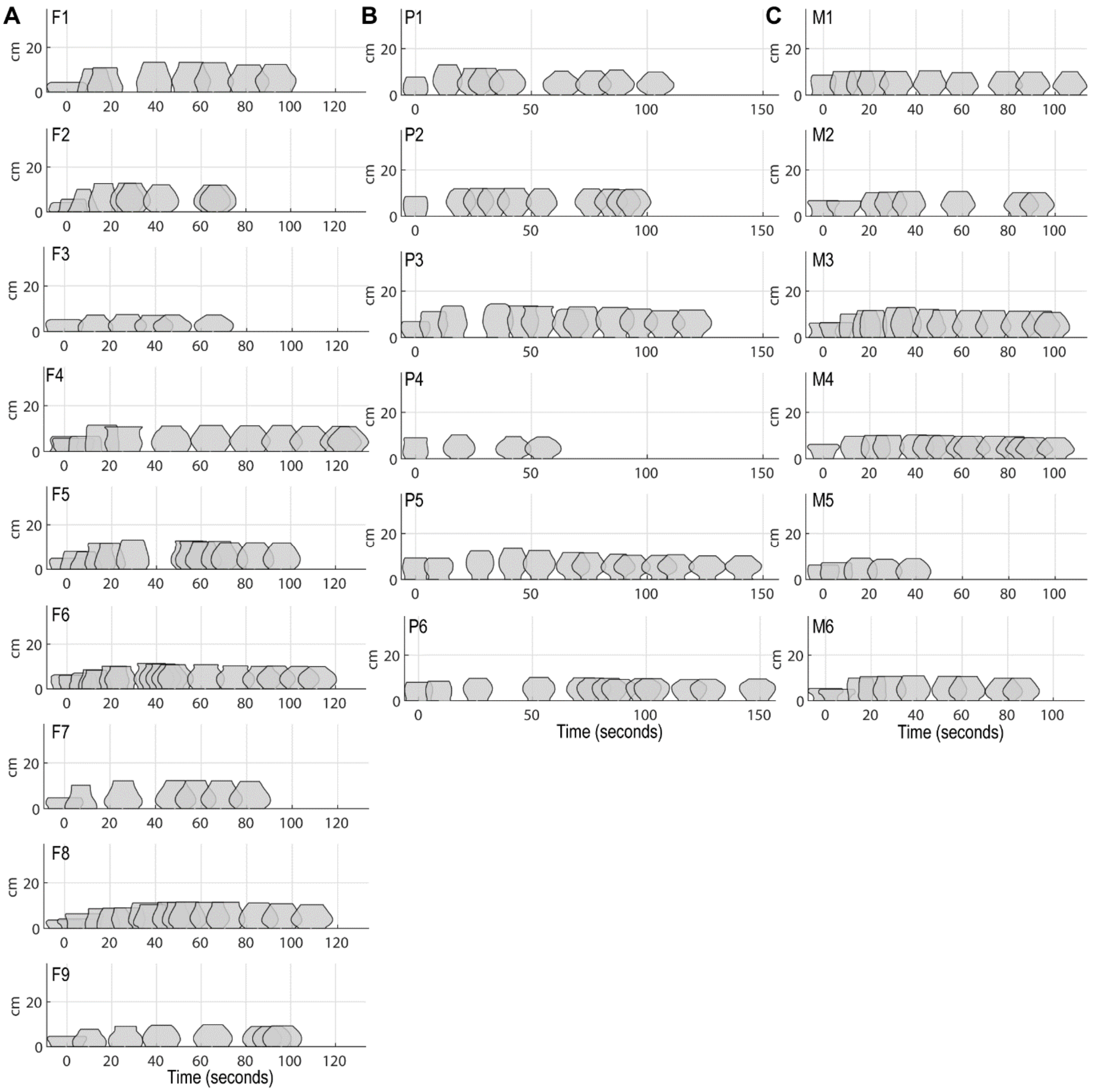


**Figure S7.** Morphological development as a function of time for the last of the five trials of producing Vase using 0.75 kg of clay thrown by each of the 21 potters. (A) Prajapati potters, (B) French potters, and (C) Multani Kumhar potters. Successive outlines on the timelines represent the vessel form after each fashioning gesture of the potter, from the initial pre-formed shape (*t* = 0) up to the final vessel shape. The size scale (height) is indicated on the y-axis.

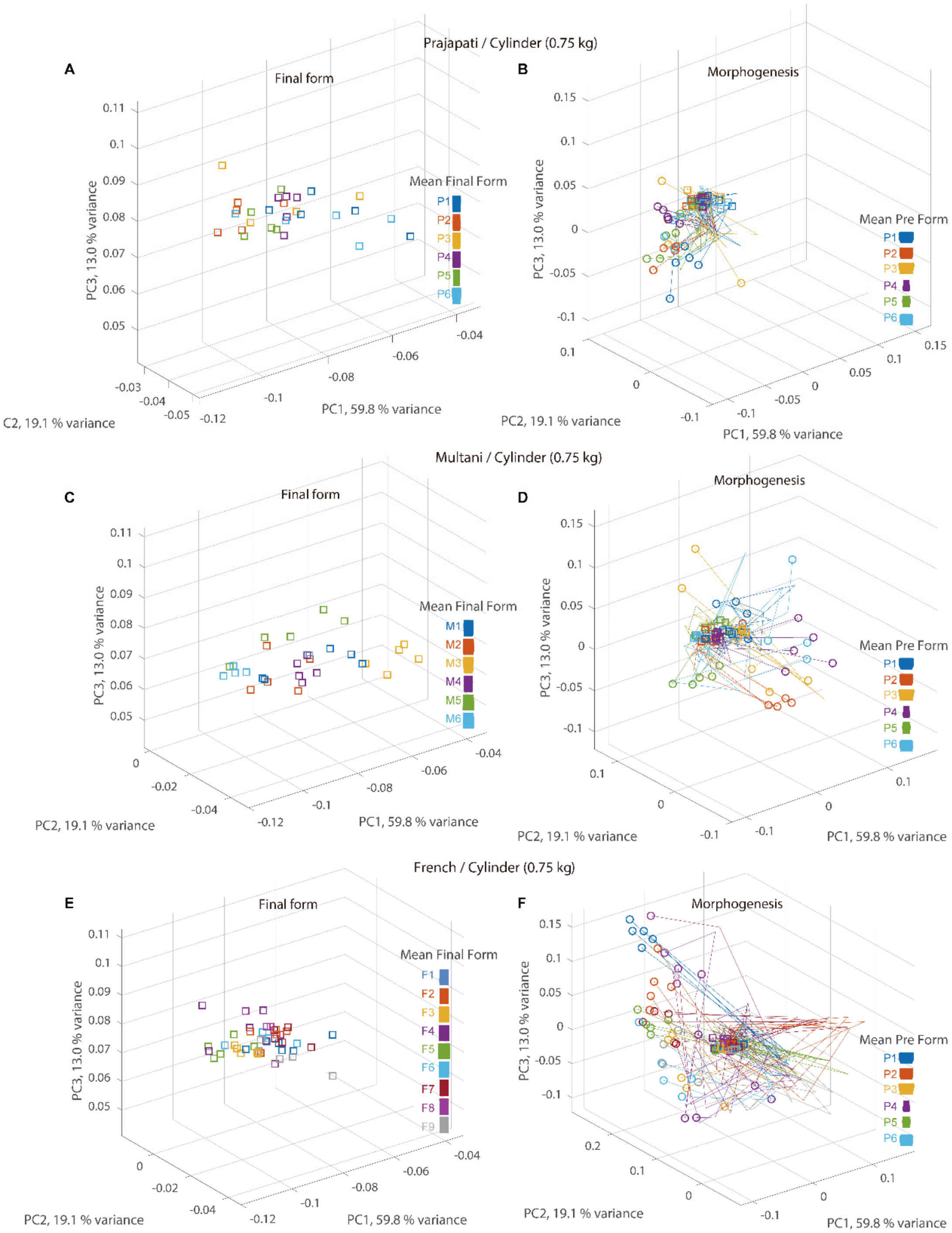


**Figure S8.** Development of the morphology of Cylinder (0.75 kg) in shape space. Right panels: Development of vessel morphology is represented as trajectories through 3D shape space, from the initial pre-formed shape (open circles) to the final shape (open squares), for vessels thrown by six Prajapati potters (B), by six Multani Kumhar potters (D) and by nine French potters (F). Individual potters are color-coded. For each potter mean initial shape (pre-form) is depicted on the right side. Left panels: Zoom on final vessel shapes (open squares) thrown by Prajapati potters (A), by Multani Kumhar potters (C), and by French potters (E). For each potter, the mean final form is depicted on the right side.

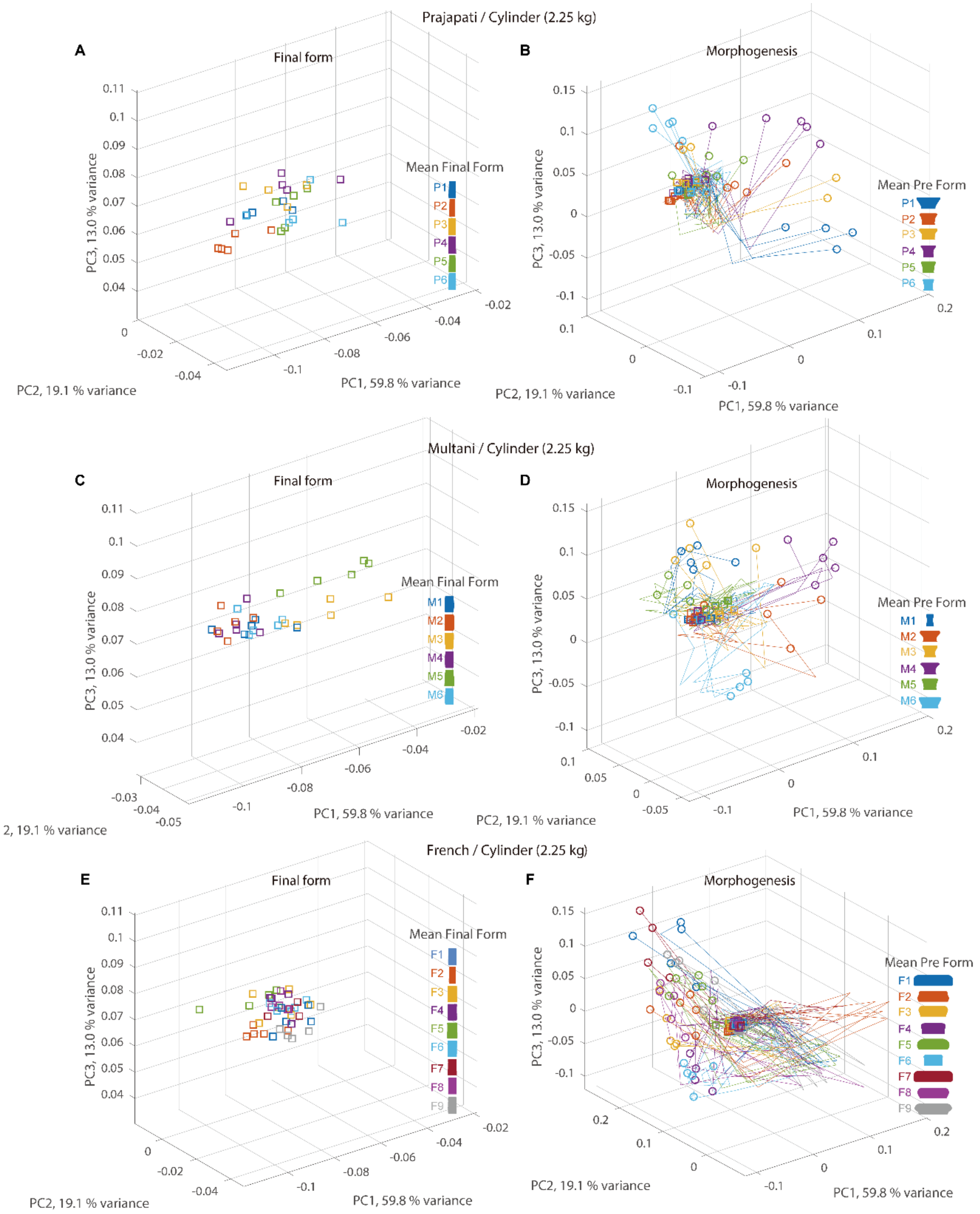


**Figure S9.** Development of the morphology of Cylinder (2.25 kg) in shape space. Right panels: Development of vessel morphology is represented as trajectories through 3D shape space, from the initial pre-formed shape (open circles) to the final shape (open squares), for vessels thrown by six Prajapati potters (B), by six Multani Kumhar potters (D) and by nine French potters (F). Individual potters are color-coded. For each potter mean initial shape (pre-form) is depicted on the right side. Left panels: Zoom on final vessel shapes (open squares) thrown by Prajapati potters (A), by Multani Kumhar potters (C), and by French potters (E). For each potter, the mean final form is depicted on the right side.

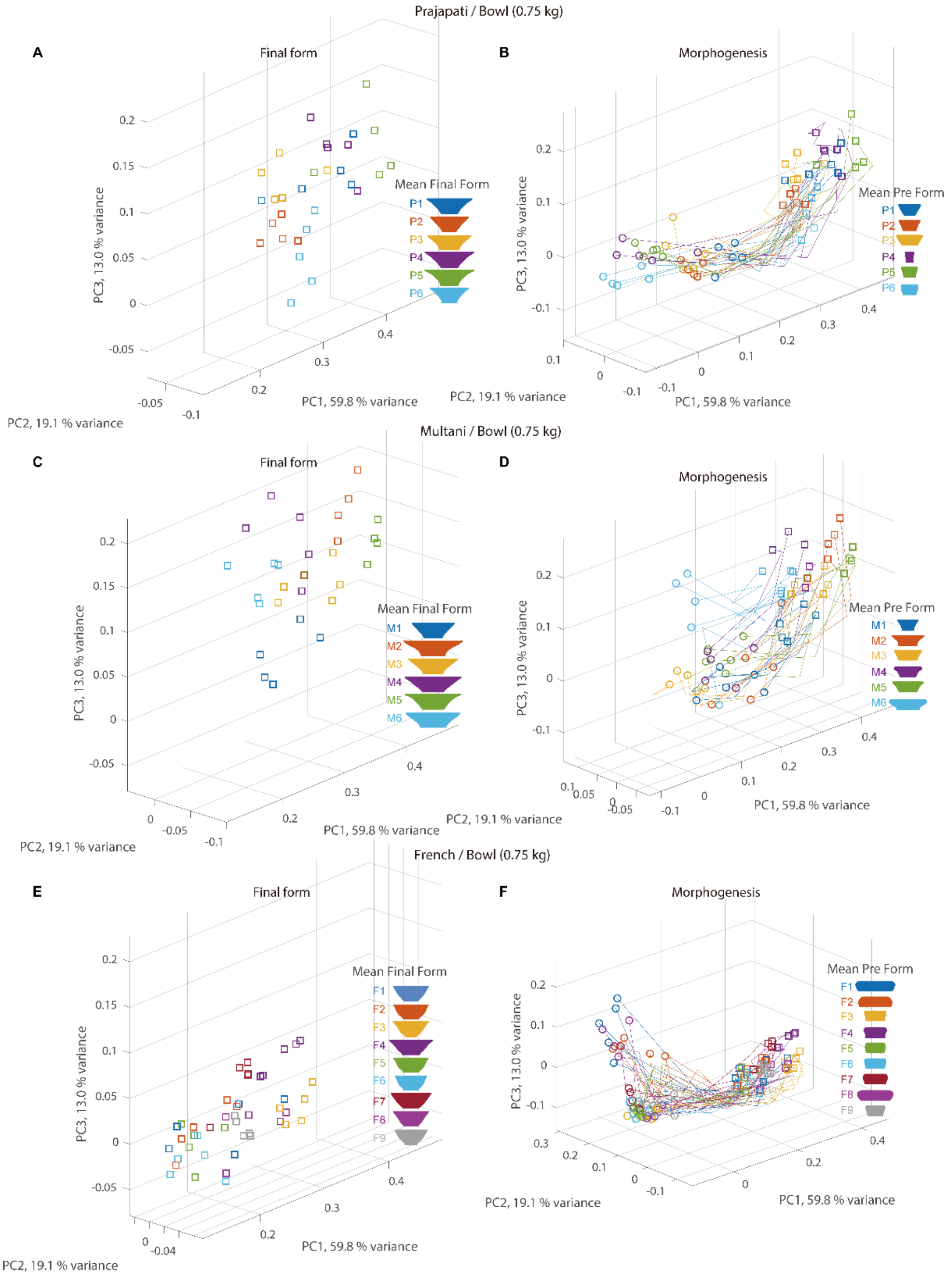


**Figure S10.** Development of the morphology of Bowl (0.75 kg) in shape space. Right panels: Development of vessel morphology is represented as trajectories through 3D shape space, from the initial pre-formed shape (open circles) to the final shape (open squares), for vessels thrown by six Prajapati potters (B), by six Multani Kumhar potters (D) and by nine French potters (F). Individual potters are color-coded. For each potter mean initial shape (pre-form) is depicted on the right side. Left panels: Zoom on final vessel shapes (open squares) thrown by Prajapati potters (A), by Multani Kumhar potters (C), and by French potters (E). For each potter, the mean final form is depicted on the right side.

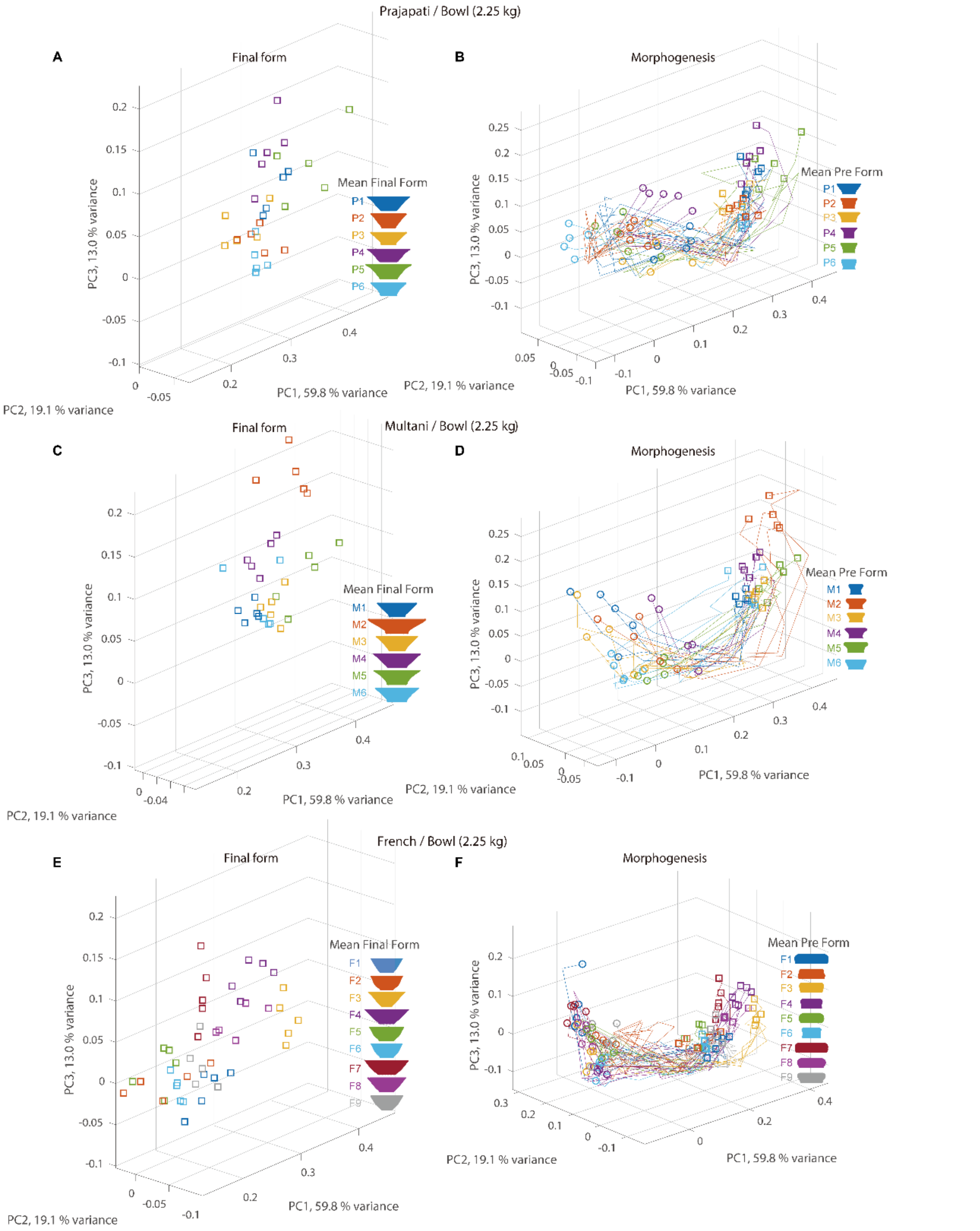


**Figure S11.** Development of the morphology of Bowl (2.25 kg) in shape space. Right panels: Development of vessel morphology is represented as trajectories through 3D shape space, from the initial pre-formed shape (open circles) to the final shape (open squares), for vessels thrown by six Prajapati potters (B), by six Multani Kumhar potters (D) and by nine French potters (F). Individual potters are color-coded. For each potter mean initial shape (pre-form) is depicted on the right side. Left panels: Zoom on final vessel shapes (open squares) thrown by Prajapati potters (A), by Multani Kumhar potters (C), and by French potters (E). For each potter, the mean final form is depicted on the right side.

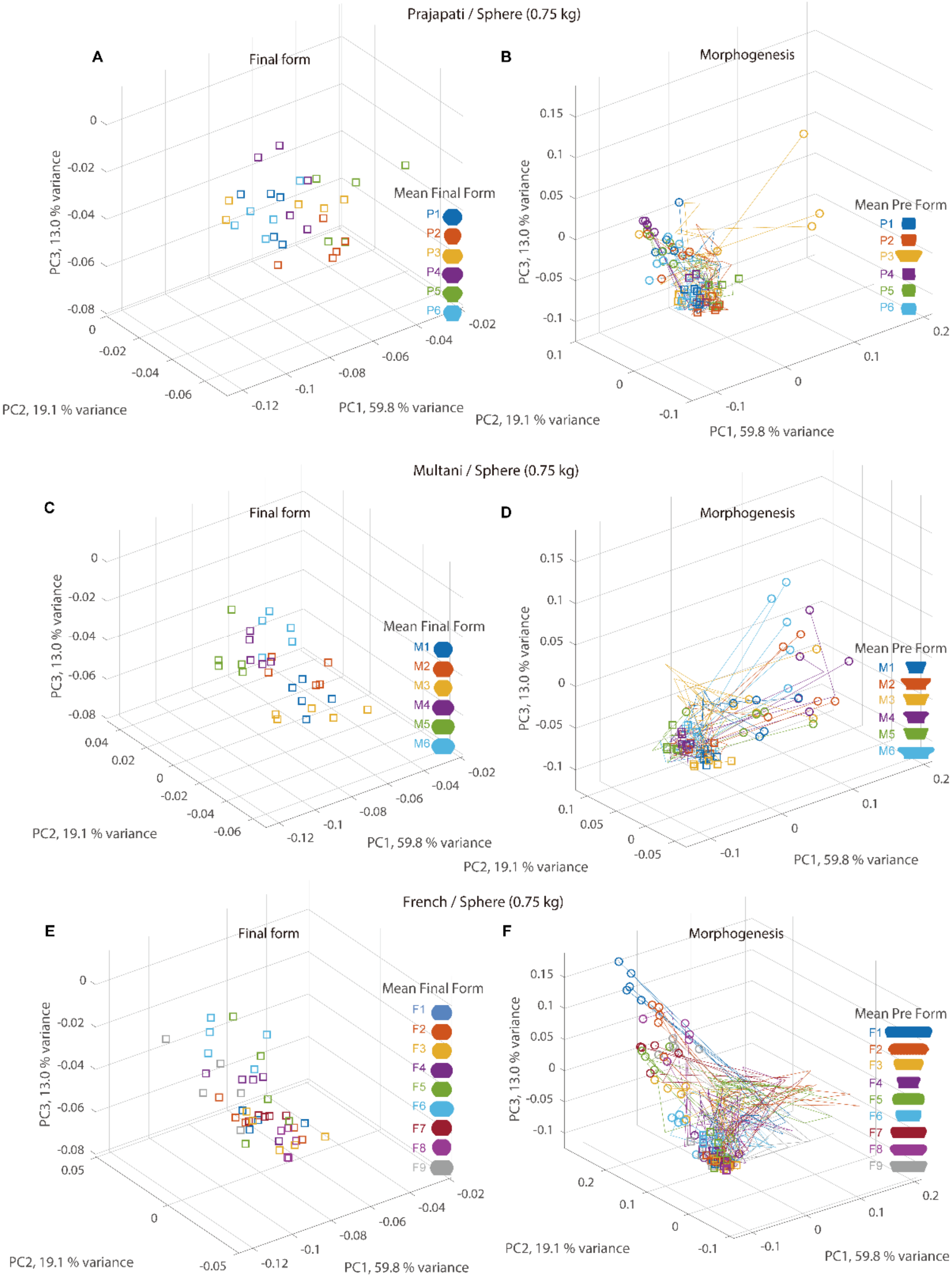


**Figure S12.** Development of the morphology of Sphere (0.75 kg) in shape space. Right panels: Development of vessel morphology is represented as trajectories through 3D shape space, from the initial pre-formed shape (open circles) to the final shape (open squares), for vessels thrown by six Prajapati potters (B), by six Multani Kumhar potters (D) and by nine French potters (F). Individual potters are color-coded. For each potter mean initial shape (pre-form) is depicted on the right side. Left panels: Zoom on final vessel shapes (open squares) thrown by Prajapati potters (A), by Multani Kumhar potters (C), and by French potters (E). For each potter, the mean final form is depicted on the right side.

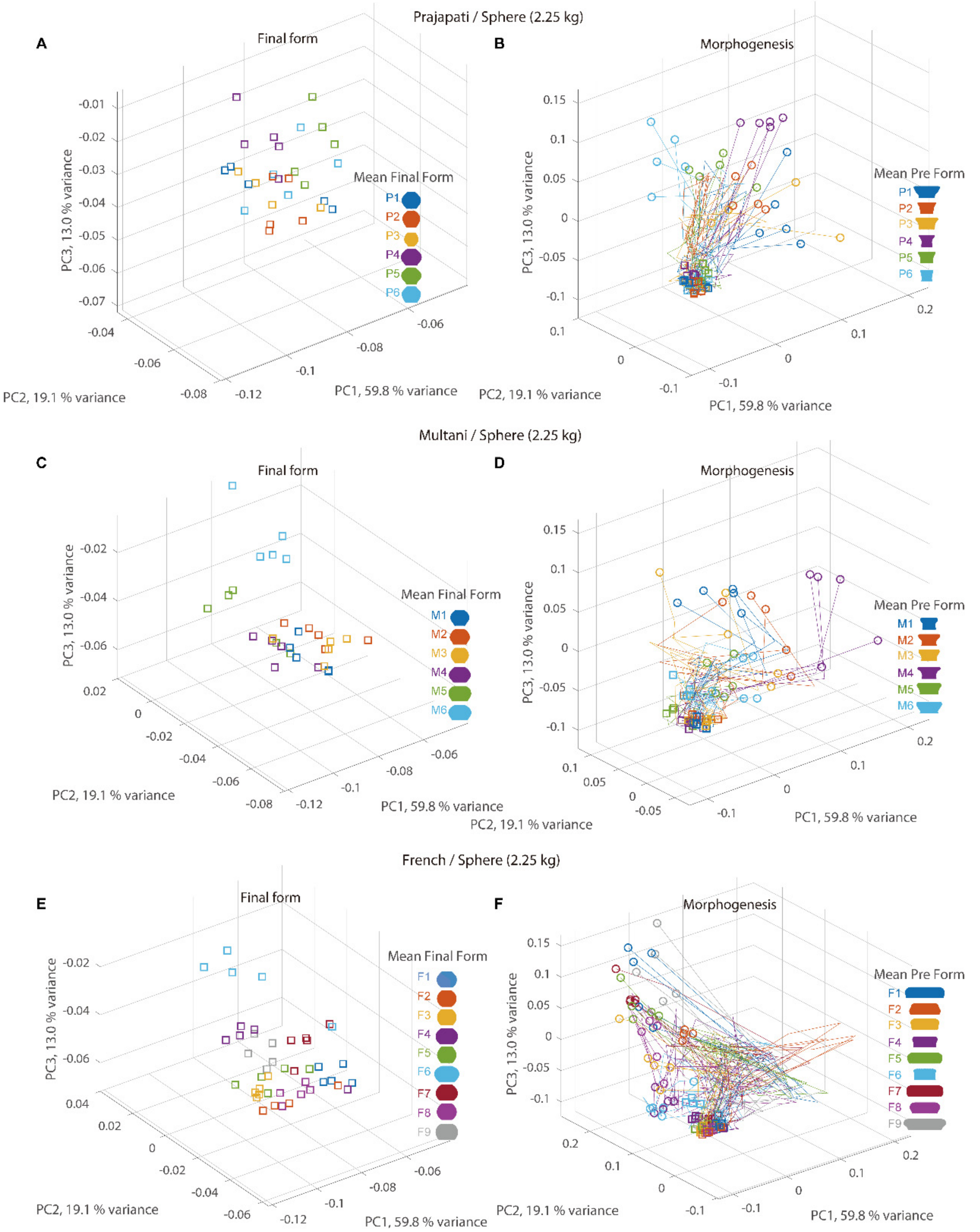


**Figure S13.** Development of the morphology of Sphere (2.25 kg) in shape space. Right panels: Development of vessel morphology is represented as trajectories through 3D shape space, from the initial pre-formed shape (open circles) to the final shape (open squares), for vessels thrown by six Prajapati potters (B), by six Multani Kumhar potters (D) and by nine French potters (F). Individual potters are color-coded. For each potter mean initial shape (pre-form) is depicted on the right side. Left panels: Zoom on final vessel shapes (open squares) thrown by Prajapati potters (A), by Multani Kumhar potters (C), and by French potters (E). For each potter, the mean final form is depicted on the right side.

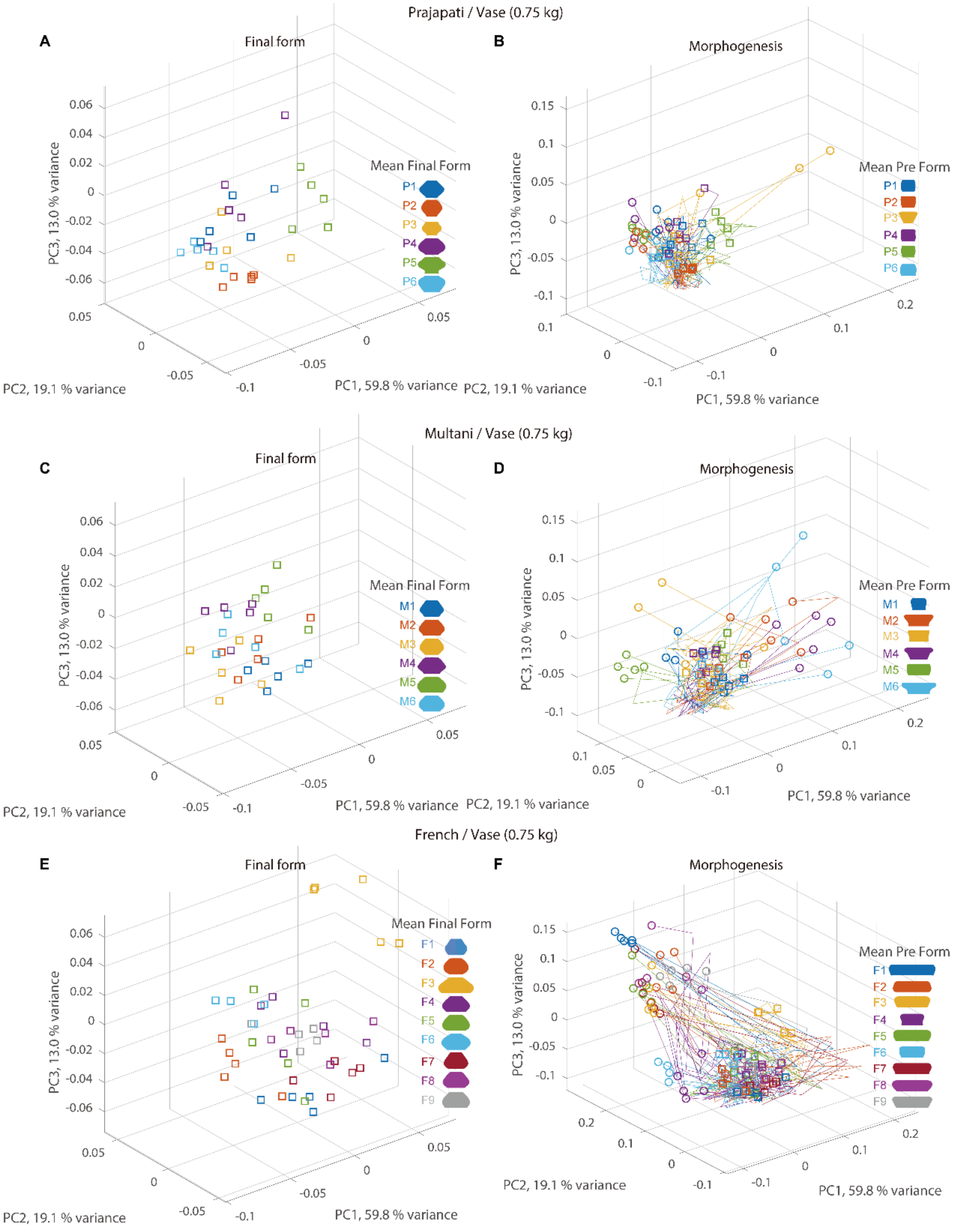


**Figure S14.** Development of the morphology of Vase (0.75kg) in shape space. Right panels: Development of vessel morphology is represented as trajectories through 3D shape space, from the initial pre-formed shape (open circles) to the final shape (open squares), for vessels thrown by six Prajapati potters (B), by six Multani Kumhar potters (D) and by nine French potters (F). Individual potters are color-coded. For each potter mean initial shape (pre-form) is depicted on the right side. Left panels: Zoom on final vessel shapes (open squares) thrown by Prajapati potters (A), by Multani Kumhar potters (C), and by French potters (E). For each potter, the mean final form is depicted on the right side.

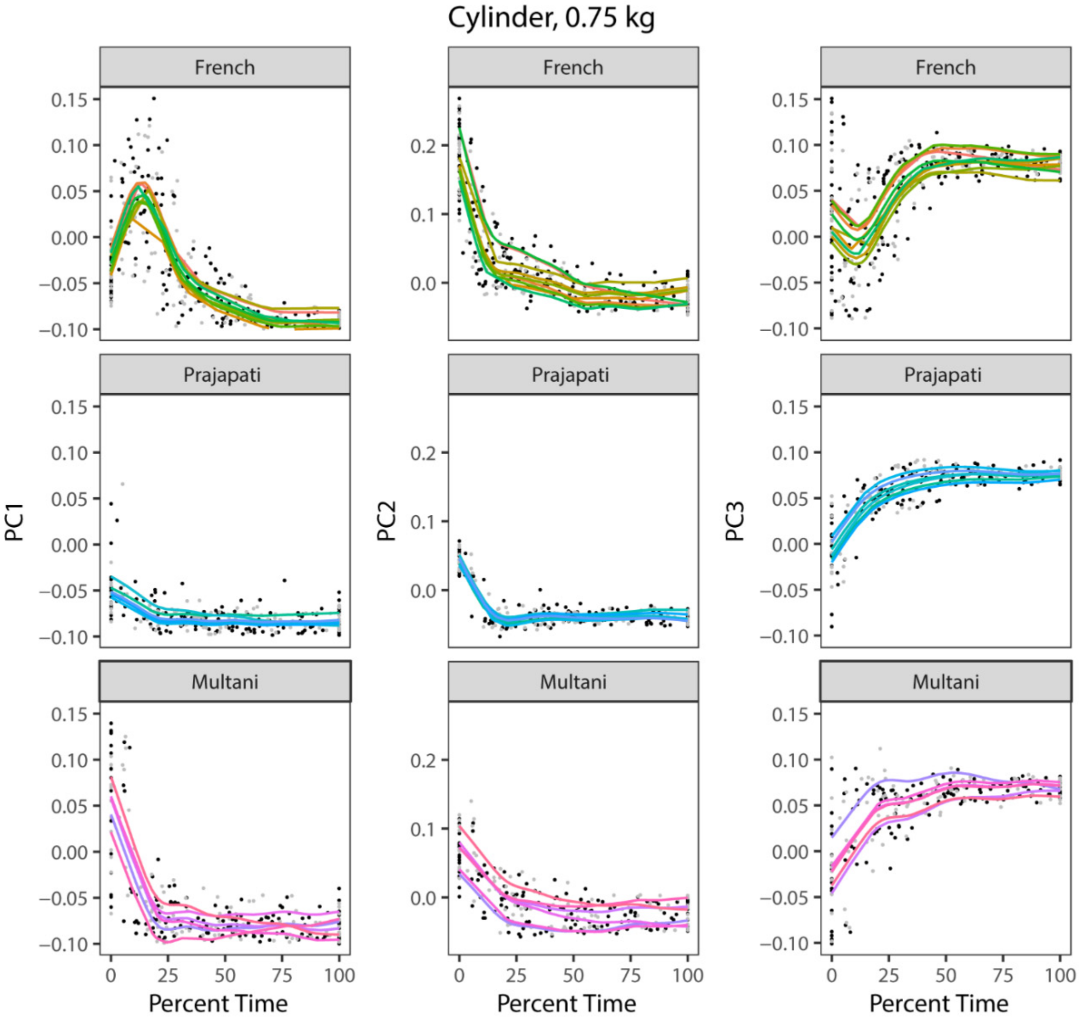


**Figure S15.** Raw data (points) and fitted generalized additive model (lines) for 3D shape space trajectory data of Cylinder (0.75 kg). Black points indicate individual trial observations in the training data (trials 1, 3, and 5), and gray points are observations in held-out trials used for model validation (trials 2 and 4) from the three communities of practice. Each line indicates predicted PC values as a function of time for each participant based on model SI (inter-community variation with similar smoothness plus random effects for individual-level intercepts and temporal variation).

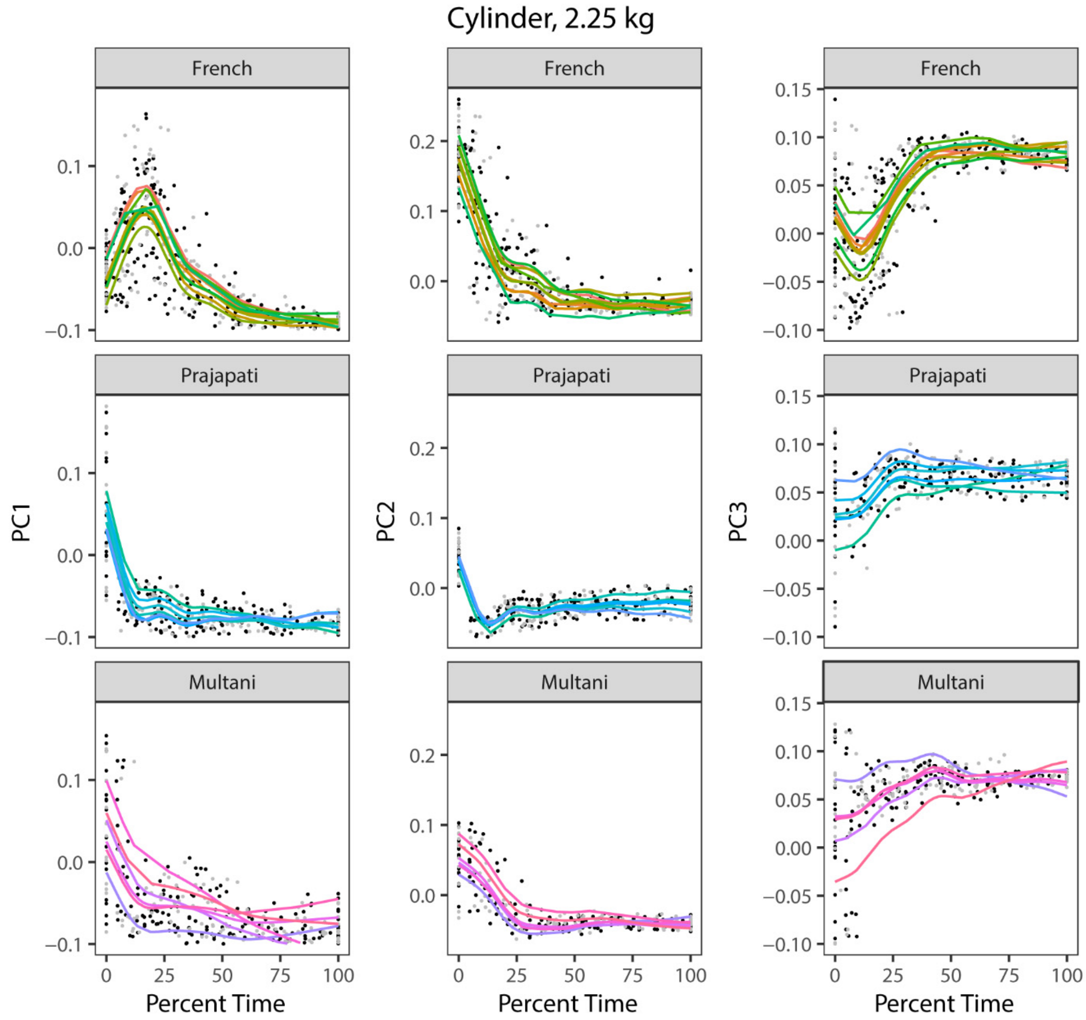


**Figure S16.** Raw data (points) and fitted generalized additive model (lines) for 3D shape space trajectory data of Cylinder (2.25 kg). Black points indicate individual trial observations in the training data (trials 1, 3, and 5), and gray points are observations in held-out trials used for model validation (trials 2 and 4) from the three communities of practice. Each line indicates predicted PC values as a function of time for each participant based on model SI (inter-community variation with similar smoothness plus random effects for individual-level intercepts and temporal variation).

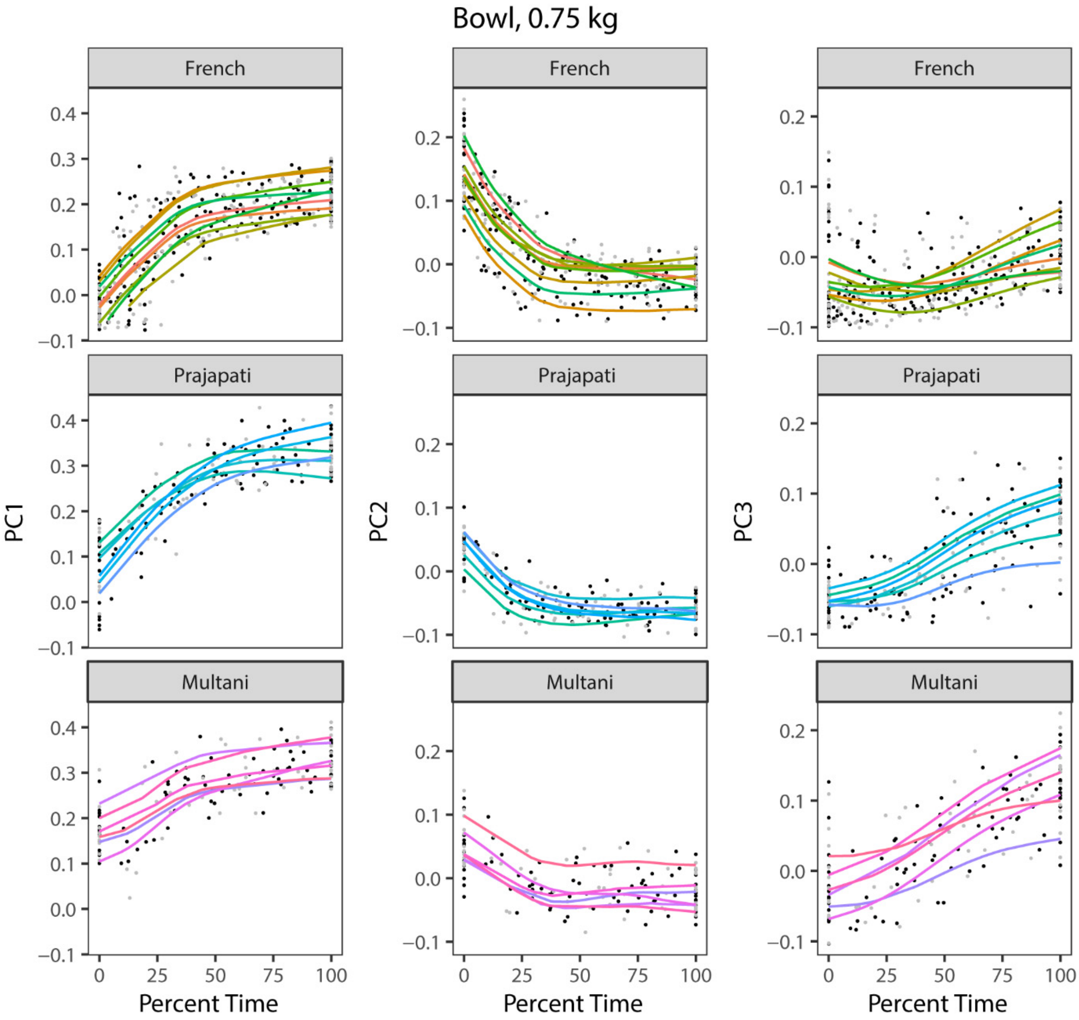


**Figure S17.** Raw data (points) and fitted generalized additive model (lines) for 3D shape space trajectory data of Bowl (0.75 kg). Black points indicate individual trial observations in the training data (trials 1, 3, and 5), and gray points are observations in held-out trials used for model validation (trials 2 and 4) from the three communities of practice. Each line indicates predicted PC values as a function of time for each participant based on model SI (inter-community variation with similar smoothness plus random effects for individual-level intercepts and temporal variation).

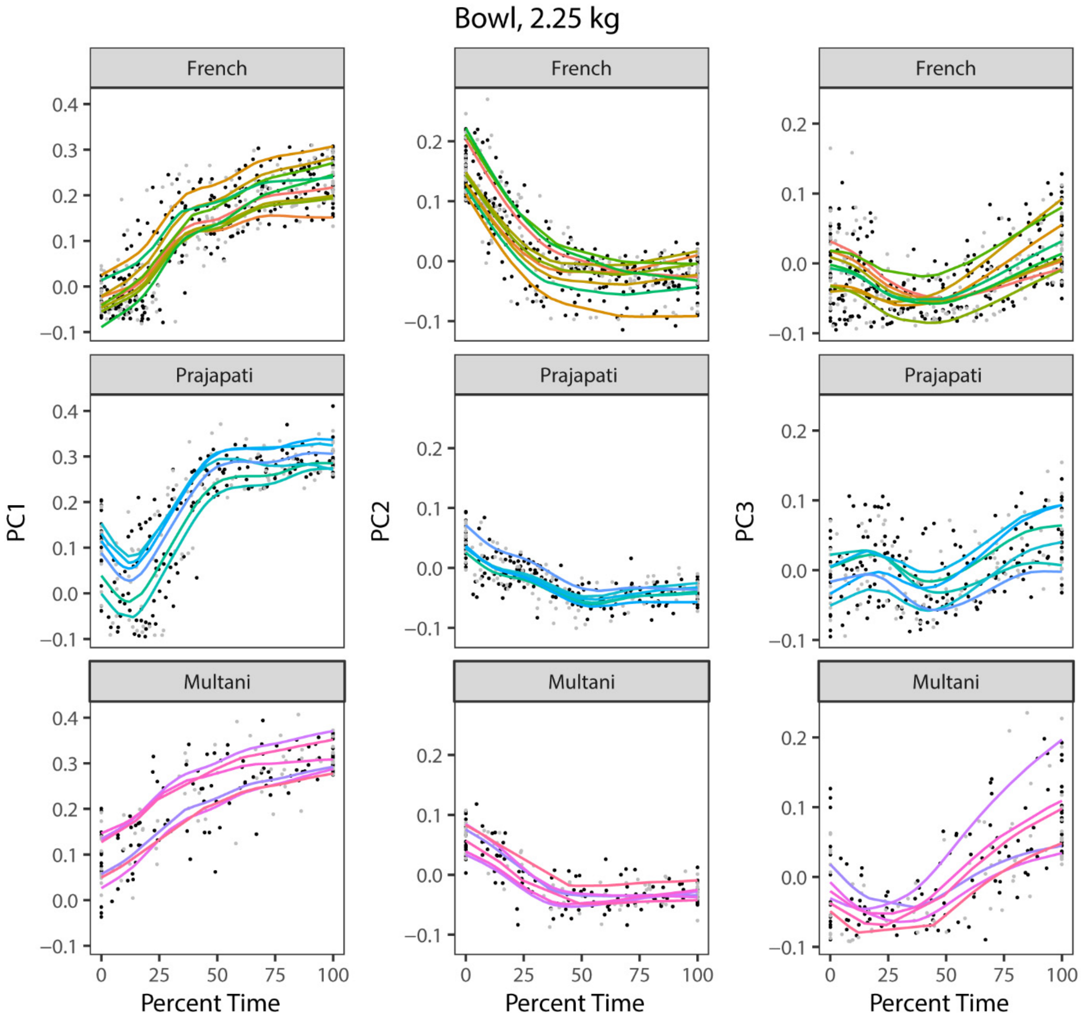


**Figure S18.** Raw data (points) and fitted generalized additive model (lines) for 3D shape space trajectory data of Bowl (2.25 kg). Black points indicate individual trial observations in the training data (trials 1, 3, and 5), and gray points are observations in held-out trials used for model validation (trials 2 and 4) from the three communities of practice. Each line indicates predicted PC values as a function of time for each participant based on model SI (inter-community variation with similar smoothness plus random effects for individual-level intercepts and temporal variation).

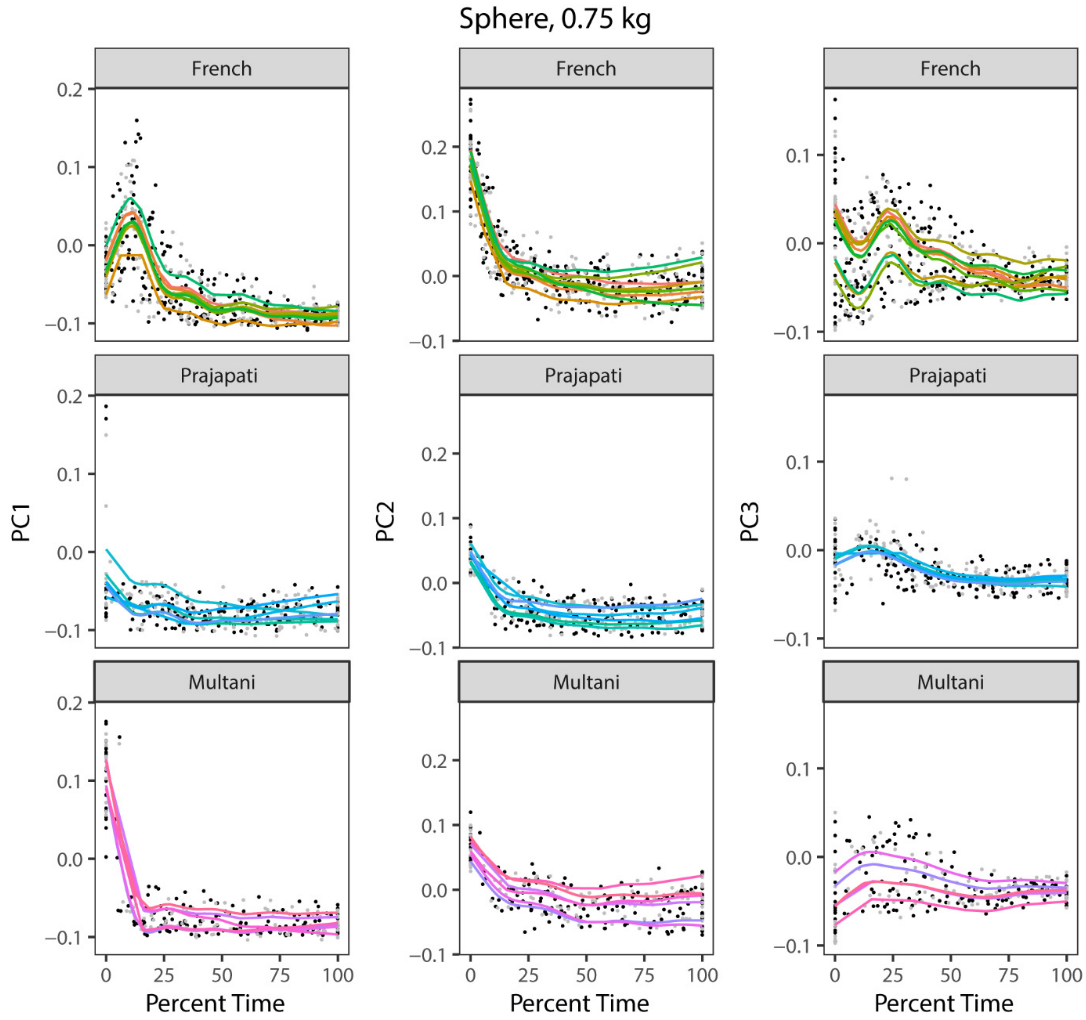


**Figure S19.** Raw data (points) and fitted generalized additive model (lines) for 3D shape space trajectory data of Sphere (0.75 kg). Black points indicate individual trial observations in the training data (trials 1, 3, and 5), and gray points are observations in held-out trials used for model validation (trials 2 and 4) from the three communities of practice. Each line indicates predicted PC values as a function of time for each participant based on model SI (inter-community variation with similar smoothness plus random effects for individual-level intercepts and temporal variation).

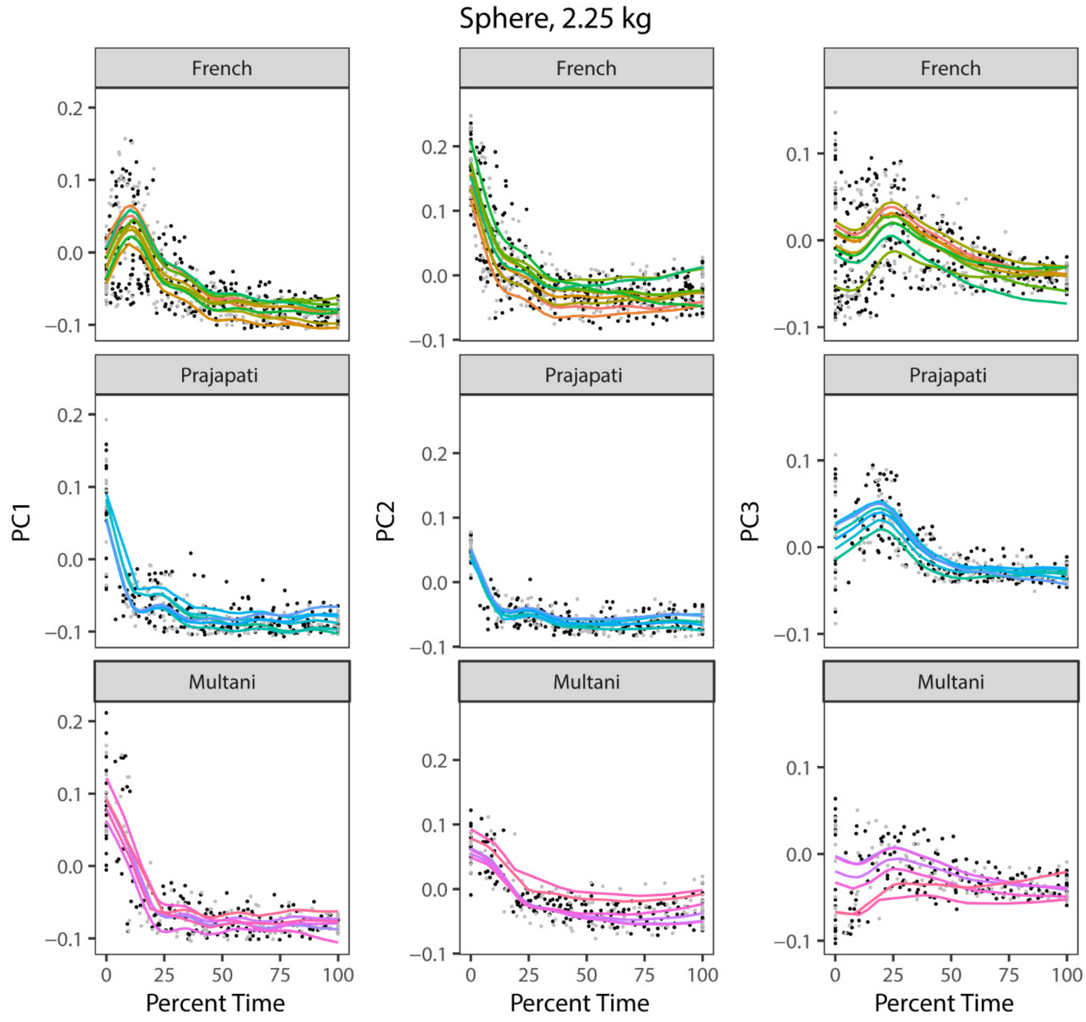


**Figure S20.** Raw data (points) and fitted generalized additive model (lines) for 3D shape space trajectory data of Sphere (2.25 kg). Black points indicate individual trial observations in the training data (trials 1, 3, and 5), and gray points are observations in held-out trials used for model validation (trials 2 and 4) from the three communities of practice. Each line indicates predicted PC values as a function of time for each participant based on model SI (inter-community variation with similar smoothness plus random effects for individual-level intercepts and temporal variation).

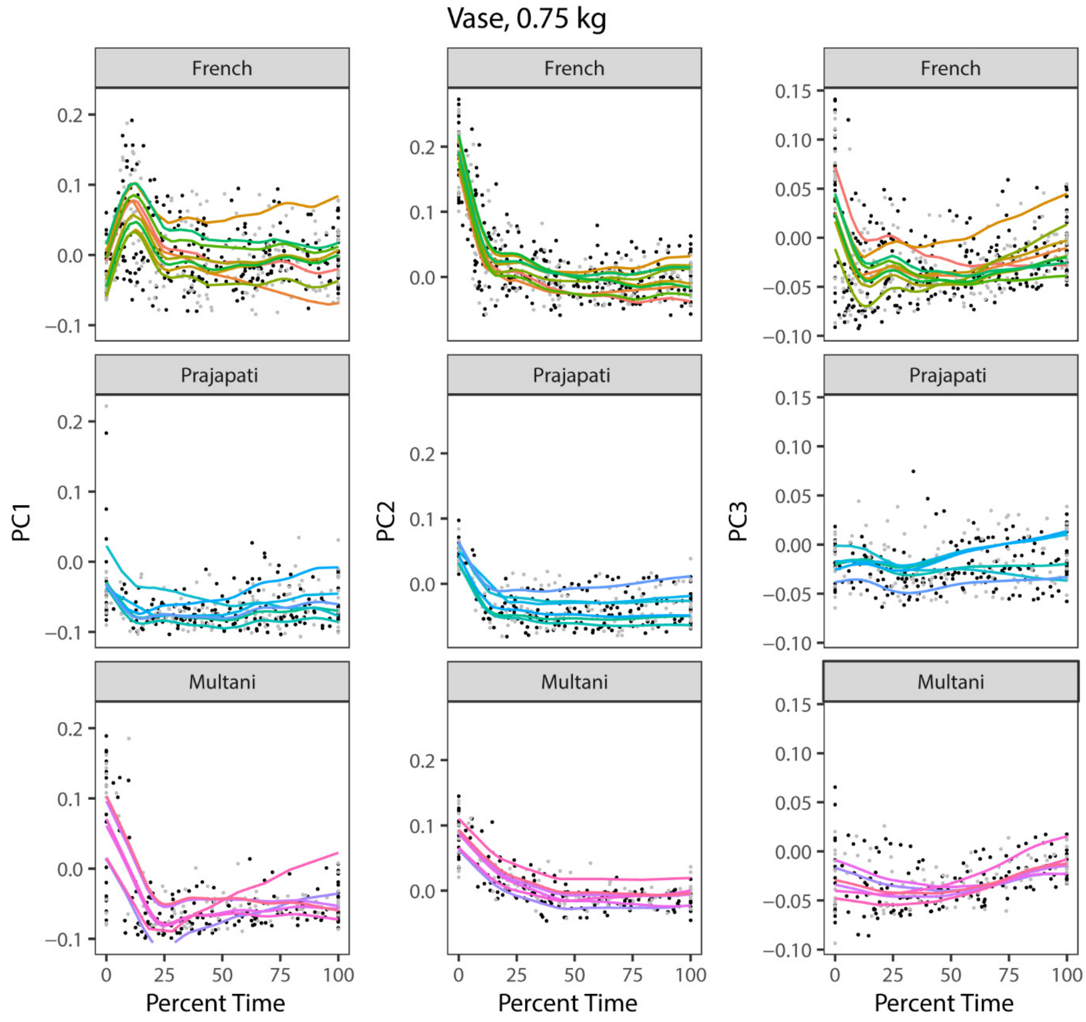


**Figure S21.** Raw data (points) and fitted generalized additive model (lines) for 3D shape space trajectory data of Vase (0.75 kg). Black points indicate individual trial observations in the training data (trials 1, 3, and 5), and gray points are observations in held-out trials used for model validation (trials 2 and 4) from the three communities of practice. Each line indicates predicted PC values as a function of time for each participant based on model SI (inter-community variation with similar smoothness plus random effects for individual-level intercepts and temporal variation).

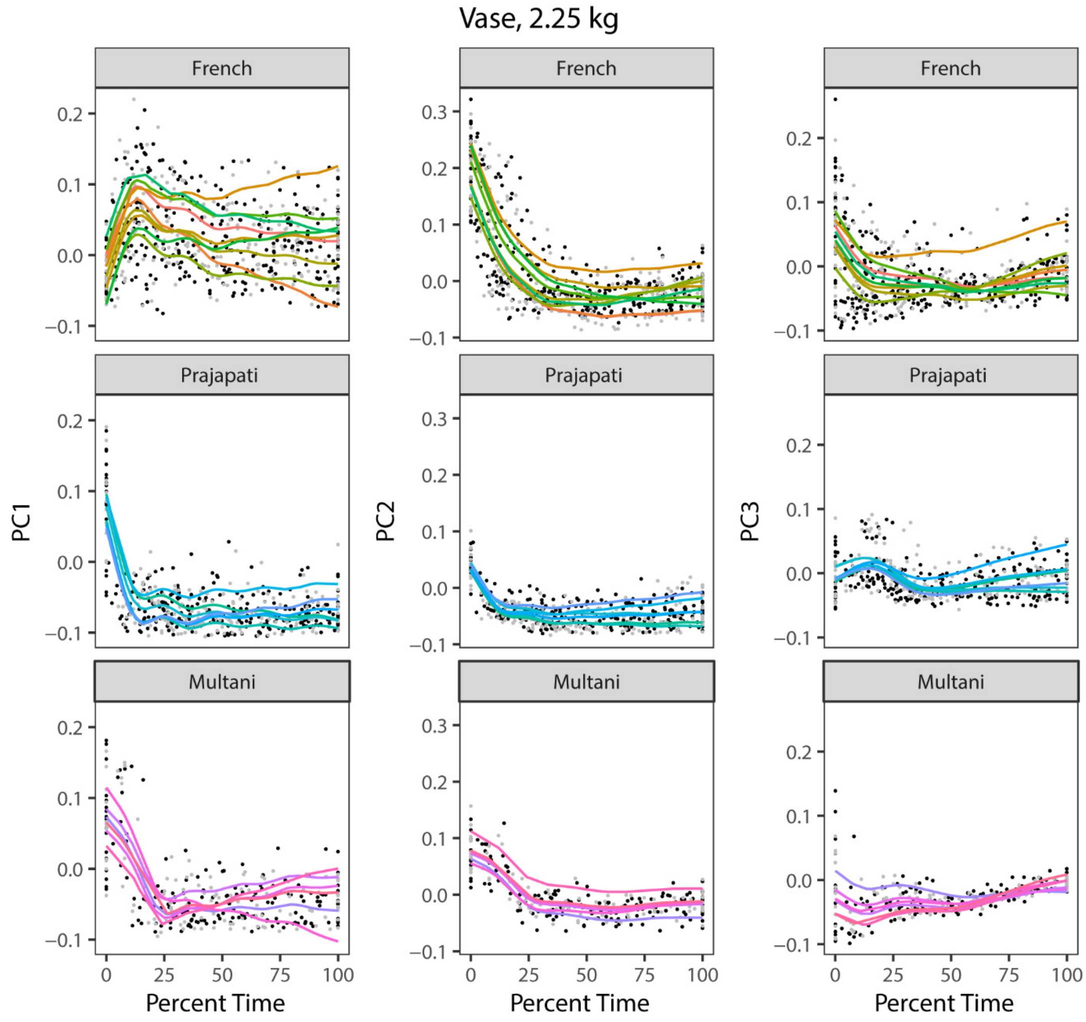


**Figure S22.** Raw data (points) and fitted generalized additive model (lines) for 3D shape space trajectory data of Vase (2.25 kg). Black points indicate individual trial observations in the training data (trials 1, 3, and 5), and gray points are observations in held-out trials used for model validation (trials 2 and 4) from the three communities of practice. Each line indicates predicted PC values as a function of time for each participant based on model SI (inter-community variation with similar smoothness plus random effects for individual-level intercepts and temporal variation).

**Table S1.** AIC (Akaike's information criterion) and out-of-sample predictive ability comparing GAM model fits for morphogenetic trajectories in three-dimensional shape space. Model G: A model with a single global smoother for all observations. Model S: A model with community-specific smoothers with a shared penalty without a global smoother. Model SI: A model with community-specific smoothers with a shared penalty plus random effects for individual-level intercepts and change over time. Model SI consistently exhibited best fit and best predictive performance of out-of-sample data for all vessel types, indicating community-specific as well as potter-specific variation in morphogenetic paths within the shape space.

| | | PC1 | | | PC2 | | | PC3 | | |
|---|---|---|---|---|---|---|---|---|---|---|
| Type | Model | *df* | AIC | Total deviance of out-of-sample data | *df* | AIC | Total deviance of out-of-sample data | *df* | AIC | Total deviance of out-of-sample data |
| Cylinder 0.75 kg | G | 8 | -3089 | 0.53 | 9 | -3180 | 0.43 | 8 | -3422 | 0.34 |
| | S | 26 | -3606 | 0.30 | 26 | -3878 | 0.20 | 23 | -3503 | 0.31 |
| | SI | 55 | -3693 | 0.28 | 61 | -4369 | 0.12 | 53 | -3638 | 0.27 |
| Cylinder 2.25 kg | G | 7 | -3391 | 0.84 | 9 | -3522 | 0.72 | 10 | -3806 | 0.55 |
| | S | 26 | -3843 | 0.53 | 27 | -4304 | 0.34 | 24 | -3973 | 0.46 |
| | SI | 61 | -4092 | 0.41 | 61 | -4563 | 0.27 | 59 | -4245 | 0.37 |
| Bowl 0.75 kg | G | 8 | -1478 | 1.90 | 8 | -2258 | 0.59 | 6 | -1981 | 0.88 |
| | S | 16 | -1962 | 0.91 | 17 | -2584 | 0.35 | 13 | -2243 | 0.59 |
| | SI | 51 | -2362 | 0.49 | 54 | -3134 | 0.17 | 46 | -2512 | 0.38 |
| Bowl 2.25 kg | G | 8 | -2211 | 2.00 | 8 | -3223 | 0.70 | 7 | -2949 | 0.88 |
| | S | 23 | -2590 | 1.3 | 19 | -3705 | 0.42 | 18 | -3091 | 0.75 |
| | SI | 58 | -3101 | 0.75 | 56 | -4405 | 0.21 | 54 | -3529 | 0.47 |
| Sphere 0.75 kg | G | 9 | -4093 | 0.63 | 10 | -4157 | 0.62 | 8 | -4534 | 0.42 |
| | S | 29 | -4740 | 0.34 | 27 | -4947 | 0.30 | 27 | -4637 | 0.39 |
| | SI | 61 | -4934 | 0.30 | 61 | -5434 | 0.21 | 61 | -5012 | 0.28 |
| Sphere 2.25 kg | G | 8 | -4505 | 0.80 | 9 | -4568 | 0.73 | 11 | -4887 | 0.54 |
| | S | 28 | -4920 | 0.55 | 27 | -5223 | 0.44 | 24 | -4988 | 0.5 |
| | SI | 61 | -5125 | 0.47 | 61 | -5669 | 0.30 | 59 | -5292 | 0.39 |
| Vase 0.75 kg | G | 8 | -3216 | 1.30 | 10 | -3804 | 0.71 | 9 | -4369 | 0.42 |
| | S | 26 | -3764 | 0.81 | 27 | -4440 | 0.38 | 25 | -4480 | 0.37 |
| | SI | 62 | -4236 | 0.55 | 59 | -4831 | 0.27 | 60 | -4820 | 0.28 |
| Vase 2.25 kg | G | 7 | -3552 | 1.90 | 9 | -4249 | 1.20 | 7 | -4819 | 0.72 |
| | S | 27 | -4464 | 0.94 | 25 | -4947 | 0.70 | 23 | -5001 | 0.62 |
| | SI | 63 | -5185 | 0.55 | 59 | -5456 | 0.47 | 58 | -5391 | 0.47 |

**Table S2.** Results (*F*, *df*, and *P*-values) for the factor Stage of the betadisper homogeneity of multivariate variance tests of the Euclidean distance from the group centroid in the full dimensional space of size-corrected Fourier coefficients, at the initial pre-formed stage, the middle stage, and the final stage, for each model vessel type thrown by the French (9 potters), Prajapati (6 potters), and Multani Kumhar potters (6 potters). For each vessel type, within-potter effects are based on five trials.

| Type | *df* | *F (Stage)* | *P* |
|---|---|---|---|
| Cylinder, 0.75kg | 2 | 218.40 | < 0.0001 |
| Cylinder, 2.25 kg | 2 | 288.09 | < 0.0001 |
| Bowl, 0.75kg | 2 | 8.00 | < 0.001 |
| Bowl, 0.75kg | 2 | 13.97 | < 0.0001 |
| Sphere, 2.25 kg | 2 | 345.11 | < 0.0001 |
| Sphere, 0.75kg | 2 | 456.80 | < 0.0001 |
| Vase 0.75kg | 2 | 106.23 | < 0.0001 |
| Vase, 2.25 kg | 2 | 183.60 | < 0.0001 |
